# A Zeolitic Imidazolate Framework-Based Antimicrobial Peptide Delivery System with Enhanced Anticancer Activity and Low Systemic Toxicity

**DOI:** 10.3390/pharmaceutics16121591

**Published:** 2024-12-13

**Authors:** Jingwen Jiang, Kaderya Kaysar, Yanzhu Pan, Lijie Xia, Jinyao Li

**Affiliations:** Xinjiang Key Laboratory of Biological Resources and Genetic Engineering, College of Life Science and Technology, Xinjiang University, Urumqi 830046, China; jjw@stu.xju.edu.cn (J.J.); 107551900870@stu.xju.edu.cn (K.K.); 990911pyz@stu.xju.edu.cn (Y.P.)

**Keywords:** ZIF-8, antibacterial peptide, drug delivery, pH response, anticancer

## Abstract

Background: The clinical efficacies of anticancer drugs are limited by non-selective toxic effects on healthy tissues and low bioavailability in tumor tissue. Therefore, the development of vehicles that can selectively deliver and release drugs at the tumor site is critical for further improvements in patient survival. Methods: We prepared a CEC nano-drug delivery system, CEC@ZIF-8, with a zeolite imidazole framework-8 (ZIF-8) as a carrier, which can achieve the response of folate receptor (FR). We characterized this system in terms of morphology, particle size, zeta potential, infrared (IR), x-ray diffraction (XRD), and transcriptome analysis, and examined the in vitro cytotoxicity and cellular uptake properties of CEC@ZIF-8 using cervical cancer cells. Lastly, we established a TC-1 tumor-bearing mouse model and evaluated its in vivo anti-cervical cancer activity. Results: The CEC@ZIF-8 nano-delivery system had favorable biocompatibility, heat stability, and pH responsiveness, with a CEC loading efficiency of 12%, a hydrated particle size of 174 ± 5.8 nm, a zeta potential of 20.57 mV, and slow and massive drug release in an acidic environment (pH 5.5), whereas release was 6% in a neutral environment (pH 7.4). At the same time, confocal imaging and cell viability assays demonstrated greater intracellular accumulation and more potent cytotoxicity against cancer cells compared to free CEC. The mechanism was analyzed by a series of transcriptome analyses, which revealed that CEC@ZIF-8 NPs differentially regulate the expression levels of 1057 genes in cancer cells, and indicated that the enriched pathways were mainly cell cycle and apoptosis-related pathways via the enrichment analysis of the differential genes. Flow cytometry showed that CEC@ZIF-8 NPs inhibited the growth of HeLa cells by arresting the cell cycle at the G0/G1 phase. Flow cytometry also revealed that CEC@ZIF-8 NPs induced greater apoptosis rates than CEC, while unloaded ZIF-8 had little inherent pro-apoptotic activity. Furthermore, the levels of reactive oxygen species (ROS) were also upregulated by CEC@ZIF-8 NPs while ROS inhibitors and caspase inhibitors reversed CEC@ZIF-8 NPs-induced apoptosis. Finally, CEC@ZIF-8 NPs also reduced the growth rate of xenograft tumors in mice without the systemic toxicity observed with cisplatin treatment. Conclusions: The CEC@ZIF-8 nano-drug delivery system significantly enhanced the anti-cervical cancer effect of CEC both in vivo and in vitro, providing a more promising drug delivery system for clinical applications and tumor management. At the same time, this work demonstrates the clinical potential of CEC-loaded ZIF-8 nanoparticles for the selective destruction of tumor tissues.

## 1. Introduction

Cancer, a formidable global health concern, has witnessed a significant decline of 32% in cancer-related mortality since 1991, thanks to advancements in early detection and efficacious treatments. However, the COVID-19 pandemic disrupted cancer diagnosis and care, with healthcare facilities closing, employment and insurance disruptions, and fear of infection persisting. Although the peak impact occurred in mid-2020, healthcare services were not entirely halted. Despite averting an estimated 3.5 million lives, cancer remains a leading cause of premature death [1]. The effectiveness of anticancer drugs is hindered by poor targeting, in vivo degradation, and severe side effects [2]. Consequently, current chemotherapy research prioritizes enhancing drug specificity and stability [3]. Promising advancements are emerging from targeted drug delivery systems, which leverage the distinct chemical and physical properties of tumors to selectively concentrate therapeutic agents, thus boosting anticancer efficacy while minimizing collateral damage [4]. These approaches encompass various chemical modifications and physical encapsulation techniques, employing nanoparticles as carriers for precision drug delivery [5].

Several nanocarrier systems with controllable drug release [6] have been approved for cancer treatment [7] or are in clinical trials, including systems in which drug release is induced by pH [8], temperature [9], redox state [10], biotin [11], light stimulation [12], or magnetism [13]. Numerous nanomaterials have also been developed for more efficient drug delivery, such as metal nanoparticles (NPs), inorganic mesoporous silica, quantum dots [14], and metal–organic frameworks (MOFs) [15]. Among them, MOFs are particularly promising controlled anticancer drug delivery vehicles due to high porosity, large surface area, tunable shape [16,17], high thermal stability, and adaptable surface functionality, properties that can improve drug biocompatibility [18] and controlled release properties [19] for more efficient tumor targeting. The zeolitic imidazolate framework-8 (ZIF-8) is one such nanoformulation constructed from tetrahedrally coordinated transition metal ions such as zinc or cobalt, with bridges formed by 2-methylimidazole (MIM) at an angle of 145° [20,21]. This structure forms a one-dimensional polymeric chain of densely interconnected lamellae maintained through compensation between π–π stacking interactions and hydrogen bonds, yielding a coordination framework with a central octahedral pattern shared by tetragonal and hexagonal faces. The large surface area of these nanoparticles confers greater drug loading capacity, biocompatibility, thermal stability, and chemical stability compared to many other MOFs [22,23,24], as well as highly selective drug delivery to the tumor based on pH sensitivity (i.e., drug release in the acidic tumor environment but not under physiological pH) [25,26]. Based on high pH sensitivity, the application of ZIF-8 nanoparticles as drug carriers has developed rapidly over the past few years, with a growing number of studies reporting successful high-capacity loading of antitumor drugs such as cisplatin [27] and doxorubicin [28,29] with enhanced antitumor efficacy and reduced side effects [26,27,28,29]. As well as composite nanoparticles own consistent stability, good biocompatibility, and protect the therapeutic drugs from chemical modification or degradation in the external environment [28,29,30].

Over the recent years, a growing body of research has exploited natural antimicrobial peptides for their potential in cancer therapy, owing to their unique attributes such as small molecular size, low immunogenicity, rapid action, inherent resistance to the development of bacterial resistance, multifaceted mechanisms [31,32,33,34], and demonstrated efficacy against a variety of aggressive malignancies [35,36]. At the cellular level, these peptides exert their anticancer effects by triggering programmed cell death (apoptosis) through the modulation of caspase activation, engagement of death receptors, alterations in the extracellular matrix, and manipulation of intracellular calcium signaling [36,37,38,39]. They also disrupt cell membranes and induce cytoskeletal damage, collectively contributing to impeding tumor proliferation and metastatic spread.

Cecropin XJ is a 37-amino acid antimicrobial peptide purified from the third stage larvae of *Bombyx mori*, a silk moth abundant in the Xinjiang Aksu Region of China [40]. Cecropin XJ belongs to the Cecropin B family of proteins with alpha-helix amphipathy. We previously reported that Cecropin XJ can dose-dependently reduce the proliferation rates of various tumor cells in vitro [41], including the growth of human Eca109 esophageal squamous cancer cells, by disrupting microtubule, microfilament, and microvillus dynamics via the downregulation of skeletal gene expression [42]. In addition, Cecropin XJ induced apoptosis of Huh7 hepatoma cells by blocking the cell cycle and depolarizing the mitochondria transmembrane potential, thereby triggering the release of cytochrome C from mitochondria into the cytoplasm and activating the caspase-3 apoptotic pathway [43]. We also found that a truncated and amidated derivative of Cecropin XJ termed CEC [44] inhibited the growth of *Staphylococcus aureus* at a lower concentration (IC_50_ of 0.25 μM) than Cecropin XJ (0.4 μM) [45], suggesting potentially greater bioactivity against tumor cells.

Despite their promising properties, the clinical implementation of antimicrobial peptides in cancer therapy faces challenges, primarily due to their non-specific effects on normal cells, causing issues like hemolysis, and rapid degradation within the body [46,47,48]. Efforts to address these limitations are focused on enhancing selectivity and specificity, such as gene therapy, immune targeting, and nanotechnology [49,50]. Among these approaches, we believe that nanocarrier-mediated delivery, particularly the encapsulation of these hydrophilic peptides within ZIF-8 in aqueous environments [51], holds significant promise for improving therapeutic outcomes. While numerous enzymes and vaccines have been successfully employed within ZIF-8 nanoparticles for enhanced catalytic activity and immunotherapy [52,53], the exploration of antimicrobial peptides’ anti-tumor efficacy using this strategy remains relatively understudied [54]. Further research is thus needed to exploit the full potential of these peptides in combination with nanocarriers for targeted cancer treatment.

Cervical cancer ranks second globally, following breast cancer, as a leading malignancy among women’s health issues [55,56], and it poses a threat to both genders, given its increasing prevalence in younger populations [57]. Its impact on individuals’ physical and mental well-being is profound. Human papillomavirus (HPV) infection is a primary cause, with over 100 subtypes classified into high-risk and low-risk groups [55,58]. The multifactorial, multigene, and multi-step etiology of cervical cancer is linked to factors like smoking, contraceptive usage, early sexual activity, irregular sex practices, and other bad habits [59]. Current treatment options for cervical cancer encompass surgery [60,61], chemotherapy [61], radiotherapy, and immunotherapy [59,62], but the efficacy of these methods falls short. Therefore, exploring novel approaches to combat cervical cancer, particularly through early detection and intervention through advancements in technology, holds immense significance for improved outcomes. Early screening, diagnosis, and treatment play a pivotal role in managing this disease effectively.

**Scheme 1:**
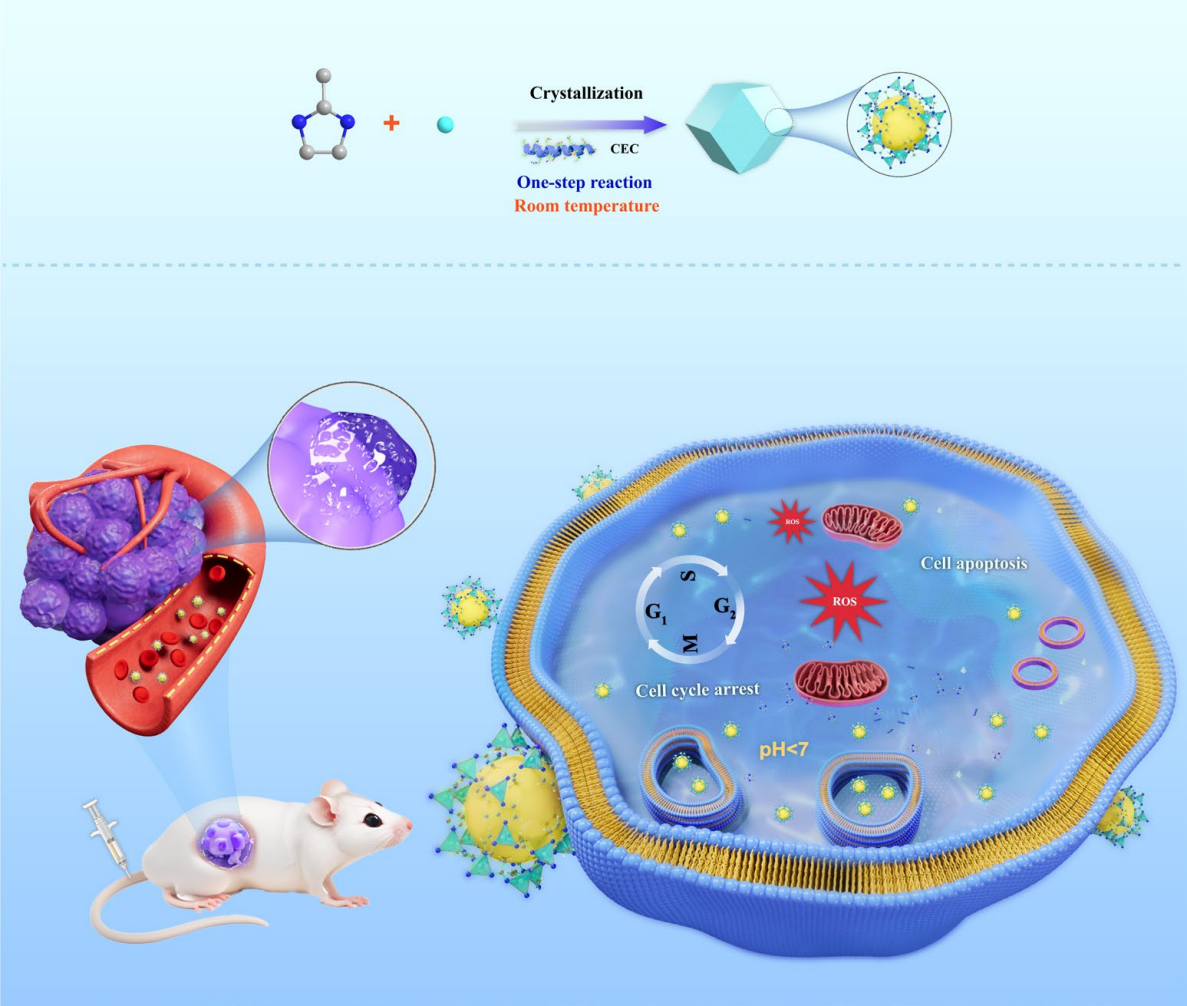
Synthesis of CEC@ZIF-8.

In the current research, ZIF-8 was selected as the nanocarrier for CEC due to its inherent pH sensitivity, substantial drug-loading capacity, thermal stability, and favorable biocompatibility. It was hypothesized that the unique porous structure of ZIF-8 would shield CEC from proteolytic degradation within the tumor microenvironment. Upon reaching the acidic tumor region, the ZIF-8 framework would dissociate, subsequently releasing the drug directly to the targeted area. To explore these possibilities, we encapsulated CEC in ZIF-8 in aqueous conditions (depicted in Scheme 1). Key properties such as particle size, stability, cytotoxicity, and gene expression alterations in cervical cancer cells were systematically assessed for the synthesized CEC@ZIF-8 nanoparticles. Our findings demonstrated that CEC@ZIF-8 nanoparticles exhibited enhanced cytotoxicity compared to the unencapsulated CEC, with significant modulation of over a thousand genes. The anti-tumor efficacy and underlying mechanisms of CEC@ZIF-8 were meticulously investigated both in vitro and in vivo. Experimentally, we observed a substantial suppression of cervical cancer cell proliferation, accompanied by cell cycle arrest and apoptosis activation via the mitochondria-mediated apoptosis pathway. Notably, in vivo tests using TC-1 cells revealed a significant reduction in tumor growth upon treatment with CEC@ZIF-8.

## 2. Materials and Methods

### 2.1. Materials and Reagents

Cecropin was synthesized by Shanghai Ketai Biotechnology (97%). Dimethylimidazole (97%) was purchased from Shanghai Yuanye Biological Technology. Zinc nitrate hexahydrate (97%), methyl alcohol, and ultra-pure water were also used for synthesis. The Annexin V-FITC Apoptosis Detection Kit was purchased from Shanghai Shengyi Biotechnology Co., Ltd. (Shanghai, China). Reactive oxygen species detection kit and mitochondrial membrane potential detection kit were purchased from Beijing Solaibao Technology Co., Ltd. (Beijing, China). Cell cycle dye and flow antibody are purchased from BD Company in China. NAC inhibitor and drug cisplatin are purchased from Shanghai Yuanye Biotechnology Co., Ltd. (Shanghai, China). Casepase 3 inhibitor was purchased from Shanghai Biyuntian Biotechnology Co., Ltd. (Shanghai, China). Phosphate buffer (PBS), trypsin, fetal bovine serum (FBS), and penicillin–streptomycin (double antibody) solutions were purchased from MRC Corporation in the United States. DMEM cell culture medium and RPMI-1640 cell culture medium were from Gibco, (Carlsbad, CA, USA). All chemical reagents were used directly without further purification.

### 2.2. Preparation of ZIF-8

Firstly, 2 g of zinc nitrate hexahydrate was dissolved in 8 mL of deionized water (solution 1), and 20 g of 2-methylmidazole solution was dissolved in 80 mL of deionized water (solution 2). Then, 40 mL of ions were dribbled into solution 1 and mixed at room temperature (14,000 rpm, 5 min). The liquid was added to solution 2 drop by drop and mixed at room temperature (14,000 rpm, 10 min). The solution produced a white suspension when mixed, which became a milky suspension after 1 min, indicating that ZIF-8 NP began to form. To ensure complete crystallization at room temperature, the solution was decomposed into a solid liquid by centrifugation (14,000 rpm, 10 mg), and the resulting white residue was rewashed 3 times with ionized water, leaving a precipitate, then frozen overnight at −80 °C and freeze-dried for storage. The dried white powder is ZIF-8 NP.

### 2.3. Preparation of CEC@ZIF-8

A large batch of CEC@ZIF-8 is described in the literature [62]. In summary, the drug-loaded CEC@ZIF-8 nanoparticles were prepared using a “one-pot stirring method”. This method is consistent with the synthesis method of ZIF-8 nanoparticles in aqueous systems. First, solution 1 (8 mL of 250 mg/mL zinc nitrate hexahydrate solution), solution 2 (80 mL of 250 mg/mL 2-methylimidazole solution), and solution 3 (40 mL of 5 mg/mL peptide (CEC) solution) were prepared. Solution 3 was dropped into solution 1 and mixed well at room temperature. The above mixture was added dropwise into solution 2 and mixed at room temperature. During mixing, the solution produced a white suspension, which turned into a milky white suspension after 1 min, indicating the formation of ZIF-8 NPs. To ensure complete crystallization at room temperature, the solution was stirred for 10 min, then decomposed solution into a solid and liquid by centrifugation (14,000 rpm, 10 min). The resulting white residue was washed with ion-free water 3 times, leaving the precipitate. It was frozen overnight at −80 °C and lyophilized for storage. The dried white powder is CEC@ZIF-8 nanoparticles.

### 2.4. Transmission Electron Microscopy Characterization

The prepared nanoparticles were suspended in an aqueous solution and dispersed by ultrasound for 10 min, then two drops (about 100 μL) of the nanoparticle samples were dropped on a microgrid. After drying, the samples were placed on a transmission electron microscope (TEM) sample rack, and the morphology and size of the synthesized nanoparticles were detected by 100 kv field emission.

### 2.5. Scanning Electron Microscopy Characterization

The nanoparticles were dipped into the conductive tape with a toothpick, and the excess was blown away with an ear suction ball. The acceleration voltage of the scanning electron microscope was 5 kV, and the morphology was observed under different multiples.

### 2.6. X Powder Diffraction Measurement

The sample powder was measured using X-ray powder diffraction (XRPD) with a Cu Kα ray (λ = 1.5418 Å). The scanning range was 5~50 degrees, and the scanning speed was 10 degrees.

### 2.7. Particle Size and Zeta Potential Detection

The particle size, particle size distribution, and zeta potential of the nanoparticles were measured at the standard room temperature of 25 °C and the scattering angle was fixed at 90°.

### 2.8. Thermogravimetric Analysis

The dry CEC@ZIF-8 and ZIF-8 NPs were subjected to thermogravimetric analysis, TGA). Conditions for thermogravimetric analysis: in an air atmosphere, from room temperature ~800 °C, with a temperature rise rate 10 °C/min measurement.

### 2.9. Infrared Spectroscopic Analysis

Infrared spectroscopic analysis was conducted using a Bruker Vertex 70 Fourier transform infrared spectroscope in the range of 500~2000 cm^−1^. The dry sample potassium bromide powder was fully mixed and then pressed to determine the infrared spectrum.

### 2.10. Determination of CEC@ZIF-8 NPs Loading and Encapsulation Rates

Drug loading and encapsulation rates were calculated according to the following equations:

Drug loading content efficiency (DLC) = NP-loaded CEC mass/CEC@ZIF-8 NP mass × 100%

Drug loading encapsulation efficiency (DLE) = NP-loaded CEC mass/total CEC mass in reaction × 100%.

### 2.11. CEC@ZIF-8 Nanoparticle Release Experiment

An equal amount (20 mg in mass) of CEC@ZIF-8NPs was dispersed into PBS solutions (5 mL) with different pH values of 7.4, 6.8, and 5.5, respectively. The drug release experiment was carried out in a 37 °C water bath (120 RPM) without light oscillation. At 0, 2, 4, 6, 8, 10, and 12 h, 0.5 mL of suspension was taken from each group of samples for centrifugation, the supernatant was used for protein quantification (using the Bradford protein assay), and the rest of the solution continued the drug release process.

Drug release rate (%) = Mass of released drug/total mass of drug loaded into carrier × 100%.

### 2.12. Hemolysis

In order to detect whether CEC has hemolysis, an in vitro erythrocyte hemolysis test was designed, conducted by comparing the hemolysis effects of equal content antimicrobial peptides and coupled nanoparticles under the same external environment. Red blood cells were derived from the jugular vein blood of two healthy (C57BL/6) mice, totaling 1 mL. The anticoagulated blood was centrifuged at 1000 rpm for 5 min, and the supernatant was discarded. The cell pellet was washed with PBS and centrifuged to remove the supernatant. After repeating this process three times, the red blood cell suspension was diluted to a concentration of 2% with PBS solution. Then, 2% red blood cell suspensions containing different concentrations of CEC@ZIF-8 and corresponding equal amounts of free CECs (5 μg/mL, 10 μg/mL, 50 μg/mL, 100 μg/mL, 150 μg/mL, 200 μg/mL) were prepared. 0.1% Triton X-100 served as the positive control, and PBS as the negative control. After gently stirring, they were treated in a 37 °C water bath for 30 min and 60 min, respectively.

### 2.13. Cell Viability Assay

The effects of CEC@ZIF-8 NPs and free CEC on HeLa cell viability were examined using the 3-(4,5-dimethyl-2-thiazolyl)-2,5-diphenyl-2-H-tetrazolium bromide (MTT) cell counting assay (Sigma, Missouri, USA). Cells at the logarithmic growth phase were seeded in 96-well culture plates at 5 × 10^3^ cells/well with 100 μL of cell medium. The medium was replaced the following day with fresh medium containing CEC@ZIF-8 NPs, equal concentrations of free CEC diluted from the stock solution, or the vehicle (negative control). The clinical antitumor agent cisplatin (35 g/mL) was used as a positive control. After 24 h, cell culture plates were centrifuged at 1200 rpm for 5 min, the supernatant was discarded, and 100 μL of MTT solution (0.5 mg/mL in PBS) was added to each well. Cells were incubated for an additional 3 h at 37 °C. Cell culture plates were then centrifuged at 1200 rpm for 7 min, and the supernatant was discarded. The formazan crystals formed from MTT by viable cells were dissolved in 200 μL DMSO, and the optical density values at 490 nm (OD490) were measured on a 96-well microplate reader (Bio-Rad Laboratories, CA, USA). All treatments were conducted in triplicate. Cell viability was calculated using the following formula:

Cell viability (%) = (Absorbance in test wells/Absorbance in negative control wells) × 100%.

### 2.14. Cell Uptake Efficiency In Vitro

When cell growth reached 80%, 2, 4, and 8 h were stimulated by CEC@ZIF-8 @NR NPs. The cellular uptake process of nanoparticles was observed using CLSM with red fluorescence emitted by Nile Red (NR) under 543 nm laser excitation. Since CEC is a membrane-breaking peptide, in order to observe whether it interacts with the cell membrane after being coated with ZIF-8, DID green fluorescent cell membrane dye was selected to stain the cell membrane through the green fluorescence generated by 664 nm laser excitation. DAPI dye solution was used to perform nuclear localization by emitting blue fluorescence under 340 nm laser excitation.

### 2.15. Clone Formation Assay

The cells were inoculated in 6-well plates at 1 × 10^3^/well and cultured continuously for 21 days. Next, 4% paraformaldehyde was fixed at 4 °C for 30 min. Crystal violet dye with a concentration of 0.5% was continuously stained for 5 min. The excess dye was washed off with PBS. Clones containing more than 50 cells were counted under an optical microscope and photographed.

### 2.16. Detection of Apoptosis

Cell apoptosis and cell death were detected using the Annexin V-FITC/propidium iodide (PI) Apoptosis Detection Kit (YEASEN, Shanghai China). HeLa, SiHa, and TC-1 cells were cultured in 60 mm cell culture dishes at 2.5 × 10^5^ cells/dish and were allowed to grow in 5 mL of culture medium. After cell attachment, the HeLa, SiHa, and TC-1 cells were treated with CEC@ZIF-8 and free CEC at various concentrations. All cells were harvested and stained with the Annexin V-FITC/propidium iodide (PI) Apoptosis Detection Kit (YEASEN, Shanghai, China) according to the manufacturer’s instructions. In some experiments, cells were pretreated with 10 mM N-acetyl-L-cysteine (NAC) and Z-VAD-FMK (FMK) for 1 h and then treated with CEC@ZIF-8 for 24 h to detect the apoptosis. Samples were analyzed using flow cytometry (BD FACSCalibur, Singapore).

### 2.17. Detection of Cell Cycle

Propidium iodide (PI) was used to analyze the DNA content in each stage of the cell cycle. Flow cytometry (FCM) was employed to conduct cell cycle analysis. The cells were cultured in 60 mm cell culture dishes at 2.5 × 10^5^ cells/dish in RPMI 1640 medium supplemented with CEC@ZIF-8 and cisplatin for 24 h. Cells were collected and washed with PBS, then fixed with 70% (*v*/*v*) ice-cold ethanol for 30 min at 4 °C. After washing twice with 5 mL PBS, cells were stained with 0.3 mL PI for 30 min at 37 °C. Samples were analyzed using flow cytometry (BD FACSCalibur, Singapore).

### 2.18. Detection of Intracellular Reactive Oxygen Species (ROS)

The level of intracellular production of ROS was measured using a fluorescent probe, 2′, 7′-dichloro-cluorescein diacetate (DCFH-DA). Cells were treated with CEC@ZIF-8 and cisplatin according to apoptosis detection for 24 h; cells were washed with PBS and stained by 10 μM of fluorescent probe DCFH-DA (Beyotime, Shanghai, China) for 20 min at 37 °C in the dark. After washing three times with PBS, the fluorescence intensity in cells was determined using flow cytometry (BD FACSCalibur, San Jose, CA, USA). In some experiments, cells were pretreated with 10 mM N-acetyl-L-cysteine (NAC) for 1 h and then treated with CEC@ZIF-8 for 24 h to detect the ROS.

### 2.19. Detection of Mitochondrial Membrane Potential (Δψ_m_)

The mitochondrial transmembrane potential was analyzed using flow cytometry with the mitochondrial membrane potential assay kit with JC-1 (Beyotime, Shanghai, China). Cells were treated with CEC@ZIF-8 and cisplatin according to apoptosis detection for 24 h. After washing twice with PBS, cells were resuspended with 300 μL of JC-1 staining solution and incubated at 37 °C for 30 min, followed by washing again with PBS twice before detection and analysis using flow cytometry (BD FACSCalibur, Singapore).

### 2.20. Animals and Ethics Statement

C57 male mice (6–8 weeks) were purchased from the Animal Laboratory Center, Xinjiang Medical University (Urumqi, Xinjiang, China), and housed in a temperature-controlled, light-cycled animal facility of Xinjiang University. All animal experiments were approved by the Committee on the Ethics of Animal Experiments of Xinjiang Key Laboratory of Biological Resources and Genetic Engineering (BRGE-AE001) and performed under the guidelines of the Animal Care and Use Committee of the College of Life Science and Technology, Xinjiang University.

### 2.21. In Vivo Tumor Study

1 × 10^5^ TC-1 cells were injected subcutaneously into the right dorsal side of C57BL/6 mice (female). After 7 days, tumor mice were randomly divided into 5 groups (4 mice per group), which were: the control group, the cisplatin group, the ZIF-8 NPs group, and CEC@ZIF-8 NPs (40 mg/kg) group, and the CEC (4 mg/kg, the same peptide content as the coupling drug) treatment group. The drug was administered through the tail vein at intervals of 2 days until the 15th day. The tumor size was measured at the specified time point, and the volume size was calculated according to the following formula:

tumor volume (mm^3^) = (length × width ^2^)/2.

At the end of the study, the survival rate of tumor mice in each group was calculated using Prizm 8.

### 2.22. Histopathological Examination

The tumor liver and kidney tissues of each group were fixed in 4% paraformaldehyde, rinsed with distilled water for 1 h, and gradient dehydration was performed with 30%, 50%, 70%, 80%, 85%, 95%, and anhydrous ethanol, and then the tissues were immersed in a mixture of anhydrous ethanol and xylene at a ratio of 1:1 for 30 min. They were soaked in xylene twice, for 1 h and 40 min, respectively. Finally, the tissues were placed in xylene and wax for 30 min and 60 min, respectively. The paraffin solidified and enshrouded the tissue and trims. A little adhesive was applied to the slide, the wax block was placed, and dripped on with steamed water overnight at 35 °C. The sections were placed in xylene and dewaxed for 10 min. They were then transferred into the mixture of xylene and anhydrous ethanol (1:1) for 5 min followed by a (100%, 95%, 85%, 70%) ethanol gradient dehydration treatment for 5 min, and rinsed. This was followed by placement in a hematoxylin dye solution for 5–15 min; hydrochloric acid alcohol separation; and the observation of clear chromatin and nuclei under a microscope. They were then washed under running water for 30 min and continued to be rinsed with distilled water; stained with 0.5% eosin solution for 5 min, followed by gradient dehydration with different concentrations of ethanol (70%, 85%, 95%, 100%) for about 3 min each time; and cleared with xylene twice for about 10 min each time; with a neutral gum seal; and the tissue changes were observed under microscope.

### 2.23. Detection of Blood Biochemical Indexes After Tumor Treatment in Mice

The blood of the mice was collected by removing the eyeballs and put into a centrifuge tube containing anticoagulants to prepare for anticoagulation. The serum was separated using a centrifuge tube, which was first placed at 4 °C for two hours, then at 37 °C for half an hour, centrifuged at 3000× *g* for 15 min, and the supernatant (serum) was collected. This was followed by the determination of red blood cell (RBC) count, hematocrit (HCT), white blood cell (WBC) count, mean corpuscular in an anticoagulation test using flow cytometry (MCH), mean corpuscular hemoglobin concentration (MCHC), platelet hematocrit (PLT), hemoglobin (HGB) concentration, mean red blood cell volume, mean red blood cell volume (MCV), alanine aminotransferase (ALT), aspartate transaminase (AST), uric acid calculus (UA), and other indicators of liver and kidney function. The detection results were statistically analyzed and compared with those of clinical control group, so as to evaluate the safety of CEC@ZIF-8NPs in healthy organism.

### 2.24. Statistics

The experimental results were processed using GraphPad Prism 8 software, and all data were expressed as mean ± standard deviation (S.D.). *p* < 0.05 is considered statistically significant.

## 3. Results

### 3.1. Construction of CEC@ZIF-8

To optimize CEC peptide loading while retaining maximal biological activity after ZIF-8 encapsulation, we compared several synthesis pathways and ultimately identified a method modified from Zhang et al. [63]. The final reaction mixture included Zn^2+^: 2-methlididazole (MIM):H_2_O at a molar ratio of 1:8:72 in water with incubation at room temperature (ZIF-8-frame synthesis). The excessive deprotonation of 2-methylimidazole allowed complexation with Zn^2+^, swiftly forming a crystal nucleus and nanometer crystalline particles around CEC. The reaction ended with neutral 2-methylimidazole combining with electropositive ZIF-8 (see Scheme 1). Consequently, an optimized synthesis scheme was established that was rapid and easy to conduct while achieving high loading efficiency and excellent stability. Pure (unloaded) ZIF-8 samples were obtained using the same incubation method without CEC.

Function of CEC@ZIF-8: schematic diagram of nanoparticle synthesis (upper portion); enhanced tumor inhibition: pH triggered, the nanoparticles disintegrate and release peptides that act on tumor cells (lower section).

### 3.2. Characterization of CEC@ZIF-8

**Figure 1:**
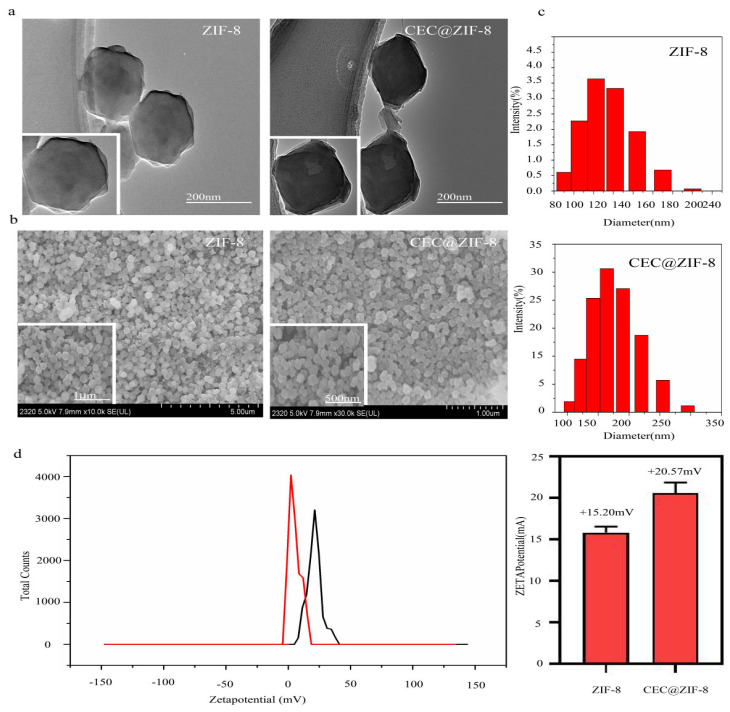
Characterization of the CEC@ZIF-8 nanoparticles and ZIF-8 nanoparticles. (**a**) TEM images of CEC@ZIF-8 and ZIF-8 nanoparticles. Scale bar, 200 nm. (**b**) SEM images of CEC@ZIF-8 and ZIF-8 nanoparticles. (**c**) DLS profile of ZIF-8, CEC@ZIF-8. (**d**) Zeta potentials of ZIF-8 NPs and CEC@ZIF-8 NPs (The red line represents ZIF-8, and the black line represents CEC@ZIF-8).

The microstructural properties of isolated ZIF-8 nanoparticles (NPs) and CEC-loaded ZIF-8 (CEC@ZIF-8) NPs were first examined using TEM and SEM. Under TEM, ZIF-8 NPs appeared as nearly rhombic dodecahedrals with a narrow size distribution, and CEC@ZIF-8 nanoparticles retained this polyhedral morphology (Figure 1a). Under SEM, both ZIF-8 NPs and CEC@ZIF-8 NPs (Figure 1b) presented as relatively uniform rhombic dodecahedral structures of 120 and 160 nm in mean diameter, respectively. Further DLS analysis The analysis of aqueous solutions indicated an average ZIF-8 particle radius of 133 ± 2.9 nm and a CEC@ZIF-8 radius of 174 ± 5.8 nm (Figure 1c), which aligns with SEM measurements and is consistent with previous studies that have reported average particle sizes ranging from 46 nm to 166 nm depending on the synthesis method and conditions. The slightly higher particle sizes may be due to the swelling of ZIF-8 and CEC@ZIF-8 in water. The size of CEC@ZIF-8 NPs was larger than ZIF-8 NPs, presumably because CEC was included.

The zeta potential of CEC@ZIF-8 was markedly more positive (Figure 1d) than that of ZIF-8 (+20.57 mV vs. +15.20 mV), suggesting successful incorporation of the peptide into the ZIF-8 framework rather than absorption on the surface. The smooth surface appearance of CEC@ZIF-8 NPs observed under SEM (Figure 1b) further supports the integration into the framework, aligning with the known characteristics of ZIF-8 materials that exhibit high porosity and chemical stability.

Energy dispersive x-ray analysis has confirmed that ZIF-8 is composed of carbon (C), nitrogen (N), and zinc (Zn), derived from 2-methylimidazole as the organic linker and zinc nitrate hexahydrate as the metal component, respectively (Figure S1a,b). This composition is consistent with ZIF-8’s known structure and its potential for various applications, as detailed in recent studies.

These elemental mapping images also reveal that the main elements (C, N, O, Zn) were randomly distributed in the ZIF-8 (Figure S1c). Identical elements were found in the dodecahedron structure of CEC@ZIF-8 (Figure S1d–f) but with a higher C/N ratio than ZIF-8 (5.0 vs. 2.4) (Figure S1b), again confirming the presence of CEC. Neither EDX spectra nor elemental mapping revealed obvious impurities in either compound.

**Figure 2:**
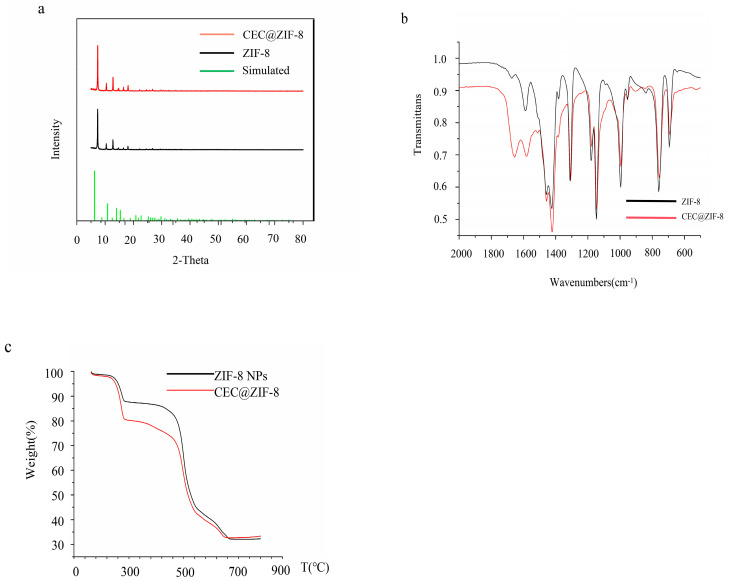
Basic properties of ZIF-8 and CEC@ZIF-8. (**a**) XRD patterns of CEC@ZIF-8, ZIF-8, and simulated ZIF-8. (**b**) FTIR spectrum of ZIF-8 and CEC@ZIF-8. (**c**) TGA curves of ZIF-8 and CEC@ZIF-8.

Powder X-ray diffraction (XRD) analysis was then performed to characterize the structures of ZIF-8 and CEC@ZIF-8. The sharp diffraction peaks observed in the XRD analysis (Figure 2a) confirmed the high crystallinity of both CEC@ZIF-8 and pure ZIF-8. All diffraction peaks of CEC@ZIF-8 were well aligned with those of pure ZIF-8, indicating that both materials retained the highly crystalline zeolite-type structures characteristic of ZIF-8. Therefore, the inclusion of CEC during the synthesis of ZIF-8 had no adverse effect on crystal formation. However, the XRD data of the resulting material of the CEC@ZIF-8 NPs and ZIF-8 NPs (Figure 2a) are identical to the simulated one, indicating the successful formation of crystal structures.

To confirm the presence of CEC in CEC@ZIF-8 NPs, Fourier transform infrared spectroscopy (FTIR) was performed. The Fourier infrared scan of ZIF-8 revealed C=N, C-N, and N-H stretch vibrations within a nitrogen heterocycle at around 1580 cm^−1^ and 500–1500 cm^−1^, respectively, indicating successful synthesis of a crystalloid, where the absorption vibration peaks at 500–1500 cm^−1^ correspond to the in-plane bending and stretching vibrations of the imidazole ring, and the absorption The peaks at 1150 cm^−1^ and 991 cm^−1^ correspond to the stretching vibrations of the C-N bonds on the imidazole ring, confirming that the ZIF-8 material maintains the integrity of the imidazole ring structure [64]. while the scan for CEC@ZIF-8 showed stretch vibrations in the same range and an additional peak at 1651 cm^−1^ (Figure 2b), which could be attributed to peptide carbonyl stretching. These analyses demonstrated that CEC molecules were present within the ZIF-8 crystals of CEC@ZIF-8 NP samples.

To evaluate the heat stability and CEC content of CEC@ZIF-8 nanoparticles, thermogravimetric analysis (TGA) was performed, increasing the temperature from room temperature to 800 °C at a rate of 10 °C per minute in an air atmosphere (Figure 2c). The ZIF-8 preparation demonstrated robust heat stability, maintaining its initial weight from 200 to 365 °C, as supported by similar studies on ZIF-8 composites. Self-ligands of ZIF-8 began to rapidly decompose at 400 °C, in line with the high thermal stability of ZIF-8, which remains stable below this temperature. However, at 600 °C, ZIF-8 undergoes complete decomposition, as evidenced by the formation of ZnO, with only carbon and zinc oxide remaining. The temperature stability curve of CEC@ZIF-8 was similar to that of ZIF-8 and exhibited three decomposition stages, including about 10% weight decrease due to CEC loss from 200 to 400 °C. However, the drug loading was 12%, as measured by the Bradford assay (Figure S2b), which is consistent with the above TGA results. Moreover, ZIF-8 was stable under physiological conditions but decomposed under acidic conditions. Therefore, the loaded drug is likely retained under a physiological environment, minimizing ectopic drug release and non-target cytotoxicity.

### 3.3. Release of CEC@ZIF-8 In Vitro

We then directly examined the effect of pH on CEC release in vitro (Figure S2a). The release kinetics of the substance were observed to be significantly faster at a pH of 5.5 compared to the physiological pH of 7.4, with rates of 50–60% versus only 6% after 12 h, respectively. This result suggests that CEC will be selectively released in the acidic environment of cancer cells. Further, ZIF-8 may help protect CEC from protease-mediated degradation. CecropinXJ is a cationic polypeptide. When it is in contact with the cell membrane, the positive charge binds to the negatively charged phospholipid, which damages the cell membrane and causes the internal substances to leak out [41,65,66]. Moreover, whether the hemolytic CEC modified by amidation has the hemolytic effect and makes a preliminary evaluation for the safety of the drug preparation delivered intravenously by CEC@ZIF-8 NPs. Therefore, hemolysis assays were performed in vitro using red blood cells (RBCs) with 0.1% TritonX-100 (100% hemolysis) as a positive control and PBS (pH 7.4) as a negative control. As shown in the Figure S3a,b, CEC@ZIF-8 NPs and free CEC exhibit minimal hemolysis at all tested concentrations, even as high as 200 μg/mL, whereas TritonX-100 lyses almost all the RBCs at a lower level (0.1%). Those results imply that the amidation antimicrobial peptide had no hemolytic effect, which indicated that chemical modification could effectively reduce the hemolytic problem of CecropinXJ, which was of great significance for its clinical application. In addition, both of them (free CEC and CEC@ZIF-8 NPs) have no hemolysis effect, and can meet the requirements of intravenous delivery therapy.

### 3.4. Intracellular Uptake and Distribution of CEC@ZIF-8

**Figure 3:**
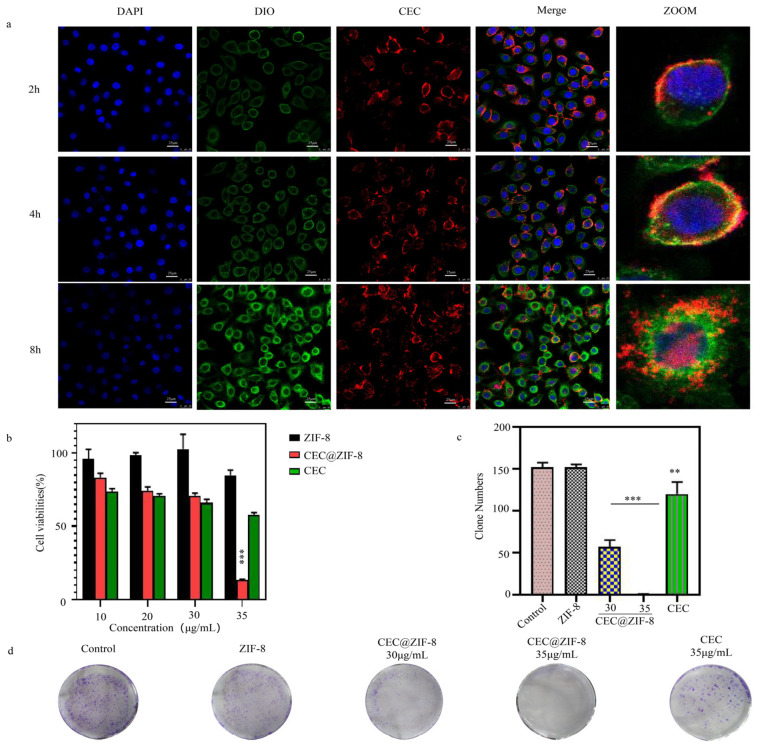
In vitro therapy effect of CEC@ZIF-8 against cervical cancer cells. (**a**) CLSM images of the distribution of drugs in HeLa cells incubated with CEC@ZIF-8 (scale bar = 25 µm). (**b**) Cell viability of HeLa cells after treatment with (1) free CEC, (2) CEC@ZIF-8, and (3) ZIF-8 for 24 h. (**c**) Differences in the number of HeLa cell clones formed by different concentrations of CEC@ZIF-8; (**d**) Statistics of the number of HeLa cell clones formed by different concentrations of CEC@ZIF-8; Compared to the control group, *** *p* < 0.001, ** *p* < 0.01.

The cellular uptake of CEC@ZIF-8NPs was then measured fluorometrically in HeLa cells by conjugation to NR (for red). For these measures, cell nuclei were stained blue by DAPI and cell membranes green by DIO. CEC-NR@ZIF-8 NPs were internalized as evidenced by a progressive increase in intracellular red fluorescence emission (Figure 3a). Initially, this red fluorescence signal was distributed mainly in the cell membrane but began to coalesce in the cytoplasm after 2 to 4 h of distribution. After 8 h of incubation, most cells exhibited large numbers of membrane vesicles at the surface resembling the apoptotic bodies described previously [67], suggesting that CEC@ZIF-8 NPs can induce tumor cell apoptosis. These findings demonstrate the efficiency of ZIF-8 NP for protecting CEC against degradation and enhancing its bioavailability for effective cellular internalization and induction of tumor cell death. Cationic polypeptides, generally rich in basic amino acid residues, interact with negatively charged phospholipids (on the cell membrane) through electrostatic attraction to form holes and break the cell membrane [65,66,68]. The charge load on the surface of tumor cells is much stronger than that of normal cells, so antimicrobial peptides generally have a strong hydrophobic effect with phospholipids on the tumor cell membrane, showing a good anticancer effect [69].

### 3.5. In Vitro Cytotoxicity Evaluation

We then examined the cytotoxicity of CEC@ZIF-8 NP, unloaded ZIF-8 NPs, and free CEC against HeLa and SiHa cells quantitatively using the methyl thiazoly tetrazolium (MTT) assay [70]. Further, the clinical anticancer drug cisplatin (35 μg/mL) was used as a positive control. Treatment of HeLa and SiHa cells with 100 to 300 μg/mL ZIF-8 NPs for 24 h was only mildly cytotoxic, with more than 80% of cells remaining viable, while 400 to 500 μg/mL ZIF-8 for 24 h was strongly cytotoxic to HeLa cells (Figure S4a). In contrast, the viability of SiHa cells was still over 80% at these high ZIF-8 doses (Figure S4c). Both CEC@ZIF-8 NPs and free CEC induced dose-dependent HeLa and SiHa cell death, but CEC@ZIF-8 exhibited significantly higher cytotoxicity towards HeLa cells, as evidenced by its notably lower half-maximal inhibitory concentration (IC50) of 32.62 ± 3.5 μg/mL compared to 65.96 ± 4.3 μg/mL for free CEC, as shown in Figure 3b and Figure S4b,d. Furthermore, the cytotoxicity of CEC@ZIF-8 NPs was significantly higher than that of unloaded ZIF-8 at comparable concentrations, as observed in studies where nanoparticles loaded with other substances exhibited improved therapeutic effects. ZIF-8 has been extensively studied for its high biocompatibility and effectiveness as a drug carrier in vivo [69]. The observed increased cytotoxicity of CEC@ZIF-8 NPs compared to free CEC is likely due to the significantly higher level of internalization (Figure 3b and Figure S4e,f) and the protection of CEC from intracellular degradation, as supported by research on ZIF-8’s properties and applications in drug delivery. The mechanism of death was then examined by monitoring changes in cell morphology during CEC@ZIF-8 NP treatment. Under control conditions, most cells were spindle- and polygonal-shaped, while treated cells became smaller and rounded (Figure S5a,b), eventually fragmenting, consistent with apoptotic death.

To investigate if CEC@ZIF-8 NPs can exert long-term therapeutic effects after single or serial exposure, we conducted clonogenic assays on HeLa cells. Both CEC@ZIF-8 NPs and free CEC inhibited the growth of HeLa cells (Figure 3c,d) as evidenced by reduced clonal numbers compared to untreated controls. Once again, clonal number was reduced to a significantly greater extent by CEC@ZIF-8 NPs than free CEC, consistent with the cell viability assay results and implying that CEC@ZIF-8 NPs have greater long-term antigrowth efficacy against these cervical cancer cell lines.

### 3.6. Mechanism Exploration of CEC@ZIF-8

**Figure 4:**
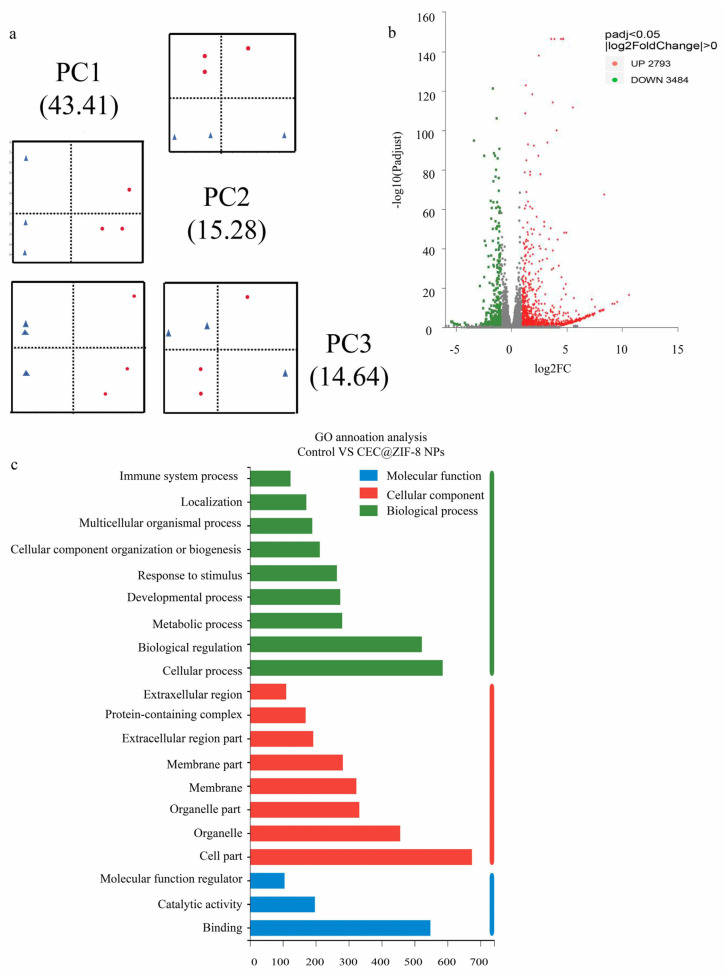
(**a**) Principal component analysis of HeLa cells based on (control) untreated control (Triangle representation) and (CEC@ZIF-8) CEC@ZIF-8 treatment groups (Circular representation). (**b**) Volcano plots to determine the DEGs of the control vs. CEC@ZIF-8 groups. (**c**) GO pathway annotation analysis of differential genes.

To examine the underlying molecular mechanisms, we first measured transcriptional changes in drug-treated and control groups and used principal component analysis (PCA) to cluster the results of different samples [71,72,73]. Briefly, in PCA, the original variables are recombined into new comprehensive variables that are unrelated (i.e., principal components), and all factors are sorted according to importance so that minor factors can be ignored, thereby simplifying the data, reducing the dimension, and facilitating graphic presentation (Figure 4a). In the PCA diagram, blue dots represent control group samples and red dots CEC@ZIF-8 NP-treated samples. As shown, all samples of the same group were clustered together in parallel directions, indicating good biological repeatability [72,73,74].

After obtaining the RNA-seq results, reads and number of counts were processed using negative binomial distribution DESeq2 software 3.20 (Majorbio Cloud provide), and differentially expressed genes (DEGs) between groups were selected according to the criteria of adjusted *p* < 0.05 and |log2FC| of 1 or higher as described [74]. Then, DEGs were screened and used to construct a volcano map with log2 (fold change) as the abscissa and -log10 (*p* adjusted) as the ordinate (Figure 4b). In this map, downregulated genes are labeled in green and upregulated genes are labeled in red. All screened DEGs are also presented in heat maps, again to better display the changes in gene expression induced by CEC@ZIF-8, with upregulated genes represented in red and downregulated genes in green. Compared to the control group, CEC@ZIF-8 treatment induced the differential expression of 1057 genes, of which 722 were upregulated and 335 downregulated.

These 1057 DEGs were then classified using the Gene Ontology (GO) database (http://www.geneontology.org/, accessed on 12 November 2024) [75] according to molecular function, biological function, and cell component. In HeLa cells, CEC@ZIF-8 NPs (Figure 4c and Figure S6a,b) induced the differential expression of genes related to the GO molecular function annotations ‘molecular function regulator’, ‘catalytic activity’, and ‘binding’, as well as the biological process annotations ‘biological regulation’, ‘metabolic process’, ‘development process’, ‘stimulus response’, ‘cell composition or biogenesis’, ‘multicellular tissue process’, and ’local immune system process’, and the cellular components annotations ‘membrane’, ‘membrane part’, ‘extracellular region part’, ‘organelle’, ‘organelle part’, and ‘extracellular region’. After enrichment analysis, the top 20 GO annotations for biological processes included ‘intracellular signal transduction’, ‘proliferation of cells positive adjustment’, ‘stimulus-response negative regulation’, ‘regulation of cells proliferation and angiogenesis regulation’, ‘motion regulation’, ‘cell migration’, ‘growth negative regulation’, and ‘cell’.

By further analyzing cell growth and death pathways in KEGG transcriptome data analysis, it was found that there were changes in 24 genes involved in the cell cycle, apoptosis, necrosis, iron death, and other specific pathways. In addition, Reactom analysis of differentially expressed genes also showed the influence of specific cell cycle pathways. Therefore, we hypothesized that cell cycle arrest and cell apoptosis might be some of the mechanisms of CEC@ZIF-8 NPs exerting anti-cervical cancer effects.

### 3.7. Mechanism Verification of CEC@ZIF-8 In Vitro

**Figure 5:**
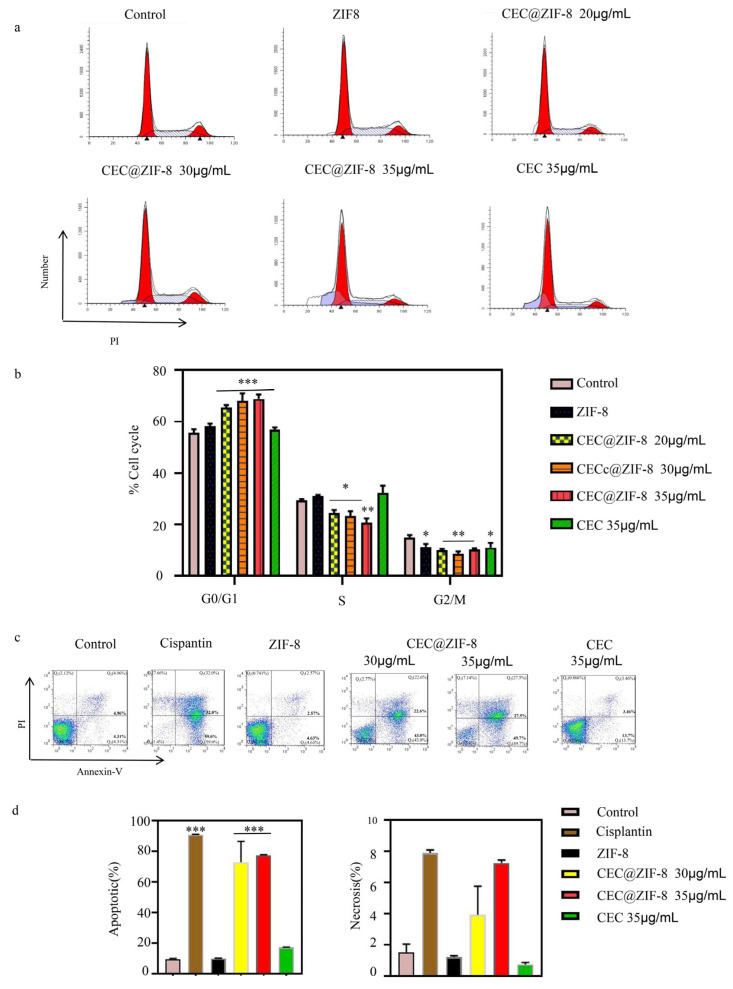
Anticancer action mechanism of CEC@ZIF-8 on HeLa cells. (**a**,**b**) Flow cytometric analysis of HeLa cell cell cycle arrest induced by CEC@ZIF-8 and free CEC. (**c**,**d**) Flow cytometric analysis of HeLa cell apoptosis induced by CEC@ZIF-8 and free CEC. Compared to the control group, *** *p* < 0.001, ** *p* < 0.01, * *p* < 0.05.

To examine the effects of CEC@ZIF-8 NPs on cell cycle progression directly, we conducted a flow cytometry analysis of cellular DNA content and distribution in CEC@ZIF-8 NP-treated HeLa cells (Figure 5a,b) as revealed by propidium iodide (PI) staining. Most treated cells were in the G1 phase, and the proportion in G0/G1 rose from 21.4% in 20 μg/mL CEC@ZIF-8 to 34.5% in 35 μg/mL CEC@ZIF-8. Further, CEC@ZIF-8 NPs induced significantly greater cell cycle arrest than free CEC, consistent with cytotoxicity assays and morphological observations of cellular apoptosis.

To further substantiate the induction of apoptosis and/or necrosis and to explore the antitumor effects of CEC@ZIF-8 NPs, we conducted Annexin V-FITC/PI dual fluorescence staining on HeLa, SiHa, and TC-1 cancer cells treated with ZIF-8 NPs, free CEC, or CEC@ZIF-8 NPs for 24 h using flow cytometry (Figure 5c,d and Figure S7a–d). The results indicated an increase in apoptotic HeLa cells from 58.5% to 63.7% when the concentration of CEC@ZIF-8 NPs was raised from 30 μg/mL to 35 μg/mL, consistent with findings from other studies that have utilized similar methods to induce and measure apoptosis in HeLa cells. The percentage of apoptotic HeLa cells was significantly higher after CEC@ZIF-8 NP treatment than free CEC treatment (Figure 5c,d). Similarly, the proportion of apoptotic SiHa cells (Figure S7c,d) increased from 17.11% in 40 μg/mL CEC@ZIF-8 NPs to 72.4% in 50 μg/mL CEC@ZIF-8 NPs, significantly higher than when treated with 50 μg/mL free CEC (24.0%), while the proportion of apoptotic TC-1 cells (Figure S7a,b) increased from 27.4% in 20 μg/mL CEC@ZIF-8 NP to 95.2% in 30 mg/mL CEC@ZIF-8 NP, which again was significantly higher than following treatment with 30 μg/mL free CEC (33%). Therefore, CEC@ZIF-8 NPs induced greater dose-dependent cell death in three distinct tumor lines compared to free CEC, consistent with MTT assay and clone formation assay results. Further, unloaded ZIF-8 was only mildly toxic, suggesting good biocompatibility.

**Figure 6:**
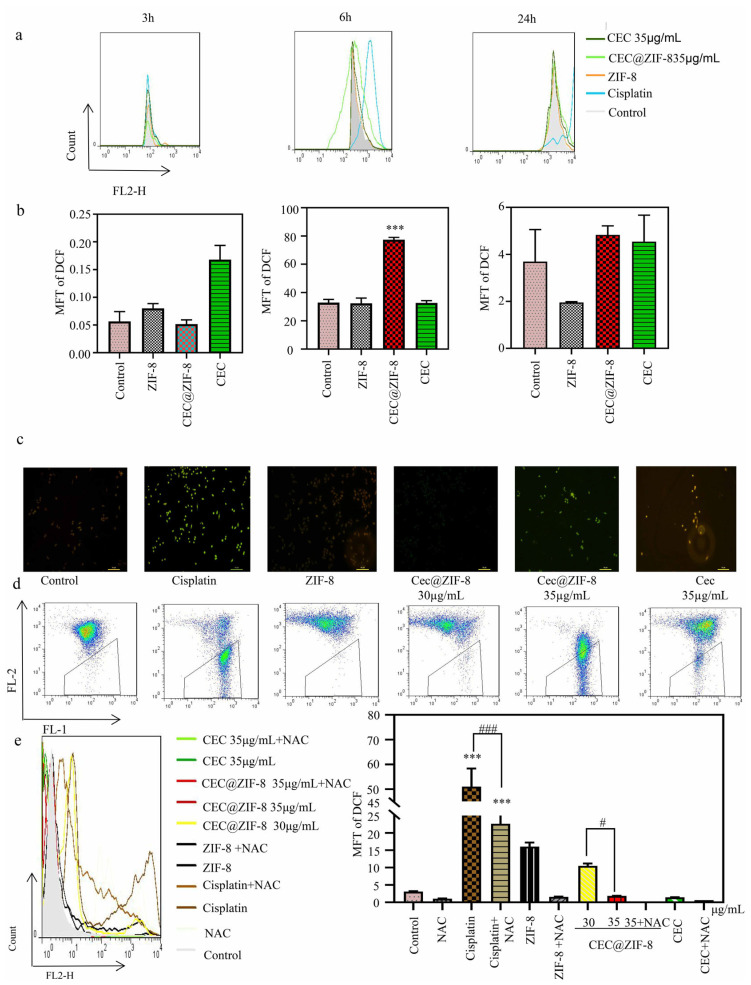
Anticancer action mechanism of CEC@ZIF-8 on HeLa cells. (**a**) ROS production in HeLa cells after CEC@ZIF-8 NPs treatment for 3, 6, and 24 h, the cells were stained with the fluorescent probe DCFH-DA and analyzed using flow cytometry. (**b**) The statistical figure. (**c**) HeLa cells were treated with 30 and 35 μg/mL CEC@ZIF-8NPs and free CEC (35 μg/mL) as the control. After 24 h, cells were treated with JC-1 dye and analyzed by inverted fluorescence microscope. (**d**) Flow cytometry was used to analyze the changes in JC-1 fluorescence. (**e**) HeLa cells were pretreated with 10 mM NAC for 1 h, and then treated with CEC@ZIF-8 NPs and CEC for 24 h. The cells were stained with the fluorescent probe DCFH-DA and analyzed using flow cytometry, and the statistical figure. Compared to the control group, *** *p* < 0.001. At the same concentration, the group treated with inhibitor was compared with the group without inhibitor treatment, # *p* < 0.05, ### *p* < 0.001.

Reactive oxygen species generated as byproducts of respiration and enzymatic reactions act as second messengers in a variety of signaling pathways [76,77]. Apoptosis can be initiated by intrinsic and extrinsic pathways as well as by excessive generation of ROS and ensuing oxidative stress. Indeed, many anticancer agents induce tumor cell apoptosis by inducing oxidative stress. To examine the contribution of oxidative stress to CEC@ZIF-8 NP-induced apoptosis of HeLa and TC-1 cells, we measured intracellular ROS generation during treatment by DCFH-DA staining and flow cytometry. As shown in Figure S8e and Figure 6a,b, CEC@ZIF-8 NPs can induce ROS production in HeLa cells and TC-1 cells at 24 h, and CEC@ZIF-8 NPs have a stronger level of ROS induction than free CEC. However, as the highest dose of HeLa cells was too strong, a large number of cells died at 24 h. Therefore, ROS were detected on HeLa cells by time gradients (3, 6, and 24 h), Figure 6a,b, and it was found that a large amount of ROS were produced at 6 h, which proved that CEC@ZIF-8 nanoparticles were time- and concentration-dependent for HeLa cells. However, the accumulation of ROS can cause a decrease in the mitochondrial membrane potential, thereby triggering mitochondria-dependent apoptosis [78,79]. To test whether apoptosis induced by CEC@ZIF-8 nanoparticles was mitochondria-dependent, ΔΨ_m_ was detected using a JC-1 probe. JC-1 probe staining and fluorescence microscopy showed that HeLa cells treated with CEC@ZIF-8 nanoparticles decreased red fluorescence and significantly increased the number of FL-1^+^ cells. At the same time, FASC results demonstrated a dose-dependent increase in the percentage of FL-2-FL-1^+^ cells, suggesting that CEC@ZIF-8 NPs effectively reduce the mitochondrial membrane potential (ΔΨ_m_) in HeLa cells. This effect was notably more pronounced than that of free CEC, as evidenced by the data in Figure 6c,d. This is consistent with findings from other studies, such as the one detailed in Reference 80, which showed that nanoparticles like NPs-PTX have a stronger inhibitory effect on A549 cells compared to their free counterparts [80].

Treatment with CEC@ZIF-8 NPs dose- and time-dependently increased ROS in both cell lines, a response inhibited by pretreatment with the ROS scavenger N-acetyl-L-cysteine (NAC). To further confirm the CEC@ZIF-8 NPs-induced levels of reactive oxygen species in cervical cancer cells, treatment with the ROS inhibitor NAC (n-acetyl-L-cysteine) showed that NAC significantly reversed the ROS production (HeLa cells) and apoptosis (HeLa cells and SiHa cells) induced by CEC@ZIF-8 nanoparticles in cervical cancer cells, suggesting that cytotoxicity is dependent in part on oxidative stress (Figure 6e, Figures S8a,b and S9a,b). Moreover, the CEC@ZIF-8 NPs-induced apoptosis (HeLa cells and SiHa cells) could be partially attenuated by the caspase inhibitor Z-VAD-FMK (Figures S8c,d and S9c,d). These results suggested that CEC@ZIF-8 NPs induced cervical cancer cell apoptosis by activating the mitochondrial apoptosis pathway.

**Figure 7:**
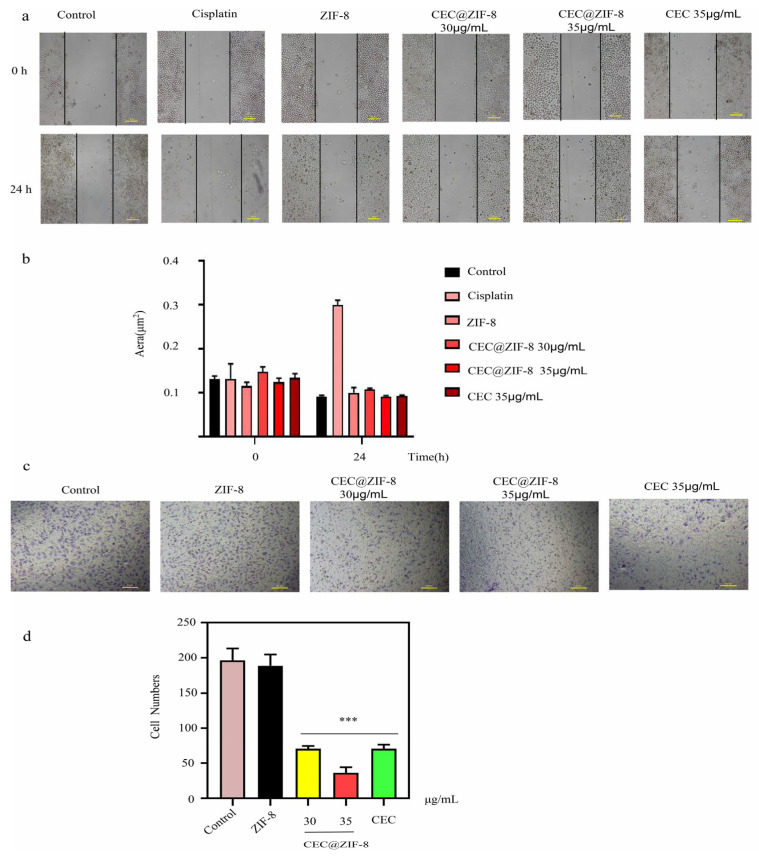
Effect of different concentrations of CEC@ZIF-8 nanoparticles on the migration of tumor cells. (**a**) HeLa cells were treated with different concentrations of CEC@ZIF-8 nanoparticles for 0 h and 24 h. (**b**) Statistical chart of the migration ability of HeLa cells. (**c**) Tumor cell invasion and statistics of HeLa cells treated with different concentrations of CEC@ZIF-8 nanoparticles for 24 h. (**d**) Statistical plot of the invasion ability of HeLa cells. Compared to the control group, *** *p* < 0.001.

Tumor migration and metastasis are the primary factors contributing to tumor progression [81]. Consequently, HeLa cells were subjected to CEC@ZIF-8 nanoparticles and free peptides, and their invasive behavior was assessed using an inverted microscope after a 24 h period. The migration of HeLa and SiHa cells was observed at 0 h and 24 h post-treatment with CEC@ZIF-8 nanoparticles and free peptides using an inverted microscope. As shown in the figure, CEC@ZIF-8 NPs inhibited the migration (HeLa and SiHa) and invasion (HeLa) of cervical cancer cells, indicating the ability of the drug to inhibit the migration and metastasis of cervical cancer cells (Figure 7a–d and Figure S10a,b).

### 3.8. Mechanism Verification of CEC@ZIF-8 In Vivo

**Figure 8:**
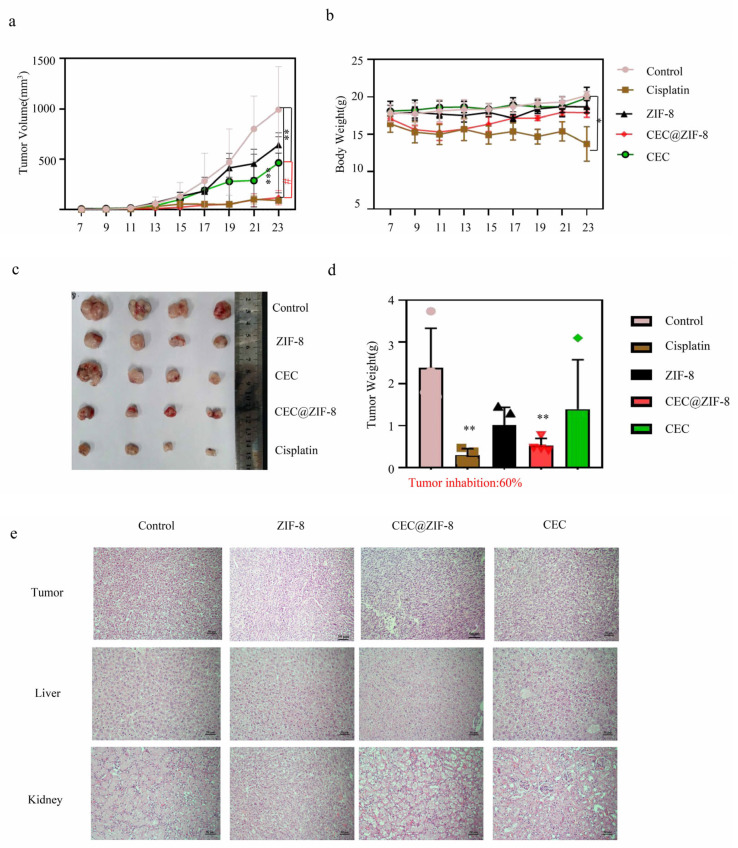
1 × 10^5^ TC-1 cells were injected into the right back of mice, and when the tumor was palpable, the mice were randomly divided into 5 groups. (**a**) The tumor volume and (**b**) body weight of the mice were measured every two days during drug treatment. Compared to the control group, * *p* < 0.05. (**c**,**d**) On day 25, the mice were sacrificed and weighed for tumor photography and weight. (**e**) Tissue sections of TC-1 tumor-bearing mice after CEC@ZIF-8NPs treatment: (1) a section of tumor tissue, (2) liver tissue section, (3) kidney tissue section. Compared to the control group, ** *p* < 0.01, *** *p* < 0.001. ZIF-8 group compared with CEC@ZIF-8 group, # *p* < 0.05.

To evaluate the antitumor efficacy of CEC@ZIF-8 NPs in vivo, we established a TC-1 tumor-bearing mouse model and assessed the impact on tumor size following treatment. Following the detection of palpable tumors, tumor-bearing mice were randomly assigned to five treatment groups (n = 4 per group) for administration of 40 mg/kg ZIF-8 NPs, 4.0 mg/kg free CEC, 40 mg/kg CEC@ZIF-8 NPs (containing 4.0 mg/kg CEC), or a control via intravenous injection every other day. As a positive control, another group of mice was administered intraperitoneal injections of cisplatin at a dose of 5 mg/kg once every 5 days. Subsequently, the mice’s body weight and tumor volume were monitored every other day for a period of 20 days, as depicted in Figure 8a,b.

Tumors grew rapidly in mice treated with the control or unloaded ZIF-8 NPs, while tumor growth was significantly inhibited by CEC and to an even greater extent by CEC@ZIF-8 NPs. Notably, there was no change in body weight in the CEC@ZIF-8 NP group, while body weight was significantly reduced by cisplatin. After 23 days of treatment and observation, mice were sacrificed, and the tumor tissues and organs isolated and weighed (Figure 8c,d). Both cisplatin and CEC@ZIF-8 NPs significantly reduced tumor weight, and average weight was significantly lower in the CEC@ZIF-8 NP treatment group than the control, free CEC, and unloaded ZIF-8 treatment groups. Further, there were no significant changes in organ weights relative to whole-body weight in any group except that the spleen weight index was significantly reduced in the cisplatin group compared to the control group, indicating that cisplatin had severe side effects (Figure S11a). Next, we examined pathological changes in tumor, liver, and kidney tissues using HE staining (Figure 8e). Compared to the Control group, the tumor tissues of CEC@ZIF-8 NP-treated mice exhibited significantly fewer nuclei and necrotic areas, while no such pathological changes were observed in liver and kidney tissues in any group.

In the tumor-forming experiment in vivo, immunohistochemistry (detection of related proteins) was also performed on tumor tissue sections. The positive staining area of Cyclin D increased gradually (immunohistochemistry), which was confirmed by in vivo experiments, indicating that cell cycle arrest is one of the mechanisms of CEC@ZIF-8NPs treatment of cervical cancer (Figure S12a).

To further examine the potential systemic toxicity of CEC@ZIF-8 NPs in vivo (Figure S12b), red blood cell (RBC) count, hematocrit (HCT), white blood cell (WBC) count, mean corpuscular hemoglobin (MCH), mean corpuscular hemoglobin concentration (MCHC), platelet (PLT) count, hemoglobin (HGB) concentration, mean corpuscular volume (MCV), and serum concentrations of kidney and liver function indicators (alanine transaminase [ALT], aspartate aminotransferase [AST], uric acid [UA], and creatinine [CREA]) were measured in blood samples obtained from the eyeball after sacrifice [82,83]. None of these measures differed between the cisplatin group and other treatment groups, indicating negligible systemic toxicity of CEC@ZIF-8 NPs and suggesting that CEC@ZIF-8 NPs can be safely and effectively used for antitumor therapy in vivo.

## 4. Discussion

Nanoparticle-based drug delivery systems for antimicrobial or anticancer peptides have garnered extensive attention and shown impressive outcomes. These nano-carriers excel at enhancing peptide specificity, cellular uptake, and permeability, as documented in several studies [84]. Various platforms, including liposomes, polymer micelles, polymer nanoparticles, gold nanoparticles, and more, have proven efficacious in encapsulating peptides [85]. Metal–organic frameworks (MOFs), a recently emerged class of hybrid organic–inorganic porous materials, offer distinct advantages due to their facile synthesis, high drug loading potential, ease of functionalization, excellent biodegradability, and benign biological interactions. Among MOFs, ZIF-8 stands out with superior attributes, such as a superior drug loading capacity, enhanced thermal stability (capable of retaining integrity up to 550 °C), chemical stability, and favorable biocompatibility, making it a preferred choice for drug delivery applications.

More importantly, this material has a sensitive pH response, which remains stable under neutral physiological conditions and starts to decompose rapidly at weak acidic sites, which can realize the controllable and accurate release of drug loading at tumor sites [86]. The antibacterial peptide (CEC) used in this study has been proven to have a certain anti-tumor effect. However, due to its short half-life and vulnerability to proteasome attack, antimicrobial peptides are limited in clinical promotion and application. The use of the CEC@ZIF-8 NPs to deliver antimicrobial peptides can effectively play the protective role of a carrier for drugs, help drugs cross the mucosal barrier, and increase the effective accumulation of drugs at the target. Similarly, Li et al. used a ZIF-8 framework material to load melittin (particle size of 100 nm, drug loading capacity of 6.9%). They intended to solve the problems of low bioavailability, poor selectivity and serious adverse reactions of some drugs. Many studies used ZIF-8 to load antitumor chemotherapy drugs with strong toxic and side effects or natural tumor treatment candidates, which provided the idea of “increasing efficiency and reducing toxicity”, built a new and efficient antitumor nanoplatform, and also broadened the application scope of MOFs in the field of biomedicine [51,87].

Simultaneously, a series of characterizations demonstrated the in vitro and in vivo anti-cervical cancer efficacy of the stable nano drug carrier system. Li et al. revealed that MLT@ZIF-8 specifically triggered apoptosis in lung cancer cells via the mitochondrial pathway [51]. Numerous studies have highlighted the ability of antimicrobial peptides to disrupt multiple functions in cancer cells, effectively inhibiting proliferation in a wide range of tumor cells, transformed cells, and solid tumors by promoting apoptosis, damaging the cytoskeleton, lysing cell membranes, suppressing metastasis, and modulating caspase expression, calcium concentration, death receptors, and the extracellular matrix [88]. Consequently, we evaluated the cytotoxic impact of the drug-loading system’s components (free CEC, empty vector, and CEC@ZIF-8) on cervical cancer cell lines, such as HeLa, SiHa, and TC-1, focusing on apoptosis. As anticipated, the drug-loaded system exhibited a dose-dependent increase in apoptosis induction, with enhanced therapeutic effects from the loaded material compared to the free form. Intriguingly, we observed a correlation between the drug-loading-induced apoptosis and intracellular ROS levels, suggesting that ROS generation plays a crucial role in apoptosis induction. Hence, ROS accumulation emerges as a pivotal target in cancer therapy strategies.

Hence, we employed zeolitic imidazolate framework-8 (ZIF-8)-based metal–organic frameworks (MOFs) to encapsulate curcumin (CEC), creating the CEC@ZIF-8 NP composite. Further functional investigations disclosed that the CEC@ZIF-8 NP could efficiently internalize into cervical cancer cells, leading to cell cycle arrest, increased production of reactive oxygen species (ROS), augmented apoptosis, suppressed tumor invasion and migration, and enhanced tumor suppression. Collectively, we have successfully designed and characterized CEC@ZIF-8 NP, demonstrating its potential anticancer mechanism, thereby enhancing CEC’s therapeutic relevance in human malignancies. These findings advocate CEC@ZIF-8 as a promising candidate for cancer management. Notably, the utilization of CEC by MOF nano-carriers highlights a cutting-edge strategy in targeting and eradicating cancer. The integration of CEC into MOFs underscores a novel approach in drug delivery, promising improved efficacy in cancer therapy.

## 5. Conclusions

The positive staining area of Cyclin D increased progressively (as detected by immunohistochemistry), further confirmed by in vivo experiments, suggesting that cell cycle arrest constitutes one of the mechanisms underlying the therapeutic effect of CEC@ZIF-8NPs on cervical cancer (Figure S11a). In contrast, unloaded ZIF-8 exhibited only mild toxicity at high concentrations, suggesting excellent biocompatibility. These CEC@ZIF-8 NPs possessed nanoscale dimensions, remarkable stability, and high loading capacity. Furthermore, the ZIF-8 shell effectively shielded CEC from intracellular degradation. Importantly, CEC@ZIF-8 NPs significantly inhibited the growth of xenograft tumors in mice without notable systemic toxicity, which was even lower than that observed with the clinical chemotherapy agent cisplatin. Bioinformatics analysis, coupled with experimental validation, revealed that these effects were mediated through mitochondria-induced apoptosis and cell cycle arrest. Flow cytometry analysis further demonstrated a greater degree of cell cycle arrest and accelerated apoptosis in the presence of CEC@ZIF-8 NPs compared to free CEC and unloaded ZIF-8 NPs. We anticipate that this pH-responsive nanodrug delivery system holds promise as a safe and effective platform for enhancing the antitumor efficacy and specificity of chemotherapeutics, including natural peptides such as CEC.

## Data Availability

The original contributions presented in the study are included in the article/Supplementary Materials, further inquiries can be directed to the corresponding author.

## Supplementary Materials

For a comprehensive understanding of the experimental data and additional analysis results, please refer to the supplementary document. The following supporting information can be downloaded at https://www.mdpi.com/article/10.3390/pharmaceutics16121591/s1: Figure S1. EDX analysis of ZIF-8/CEC@ZIF-8; Figure S2. Basic properties of CEC@ZIF-8; Figure S3. Hemolytic assay for CEC@ZIF-8 NPs and free CEC samples at different concentrations in RBC. (a) OD value at 480 nm and (b) photographs of CEC@ZIF-8 NPs samples at different concentrations in RBC, with Triton (0.1%) as a positive control (100% hemolysis, the first from the right) and PBS (pH 7.4) as a negative control (the first from the left); Figure S4. In vitro cytotoxicity of ZIF-8, free CEC, and CEC@ZIF-8 against cervical cancer cells; Figure S5. Morphology of cervical cancer cells treated with different concentrations of ZIF-8, CEC@ZIF-8, and free CEC for 24 h; Figure S6. The KEGG pathway of the difference gene is analyzed. The Reactom pathway of the different genes is analyzed. Figure S7. Anticancer action mechanism of CEC@ZIF-8 to cervical cancer cells; Figure S8. After pretreatment of cervical cancer cells with 10 mM NAC for 1 h and treatment with CEC@ZIF-8 NPs and free CEC for 24 h for Annexin V/PI staining, the samples were analyzed using flow cytometry; Figure S9. After pretreatment of cervical cancer cells with 10 mM NAC for 1 h and treatment with CEC@ZIF-8 NPs and free CEC for 24 h for Annexin V/PI staining, the samples were analyzed using flow cytometry; Figure S10. Effect of different concentrations of CEC@ZIF-8 nanoparticles on the migration of tumor cells; Figure S11. In vivo anticancer and underlying mechanisms of CEC@ZIF-8; Figure S12. Safety evaluation of CEC@ZIF-8 in TC-1 tumor-bearing mice.
