# Supplementary material for: A Zeolitic Imidazolate Framework-Based Antimicrobial Peptide Delivery System with Enhanced Anticancer Activity and Low Systemic Toxicity
Source: Pharmaceutics. 2024 Dec 13;16(12):1591. doi: 10.3390/pharmaceutics16121591 (PMC11678129; doi:10.3390/pharmaceutics16121591)
Supplement: Supplementary file 1 [file pharmaceutics-16-01591-s001.zip › pharmaceutics-3325771-supplementary.pdf]

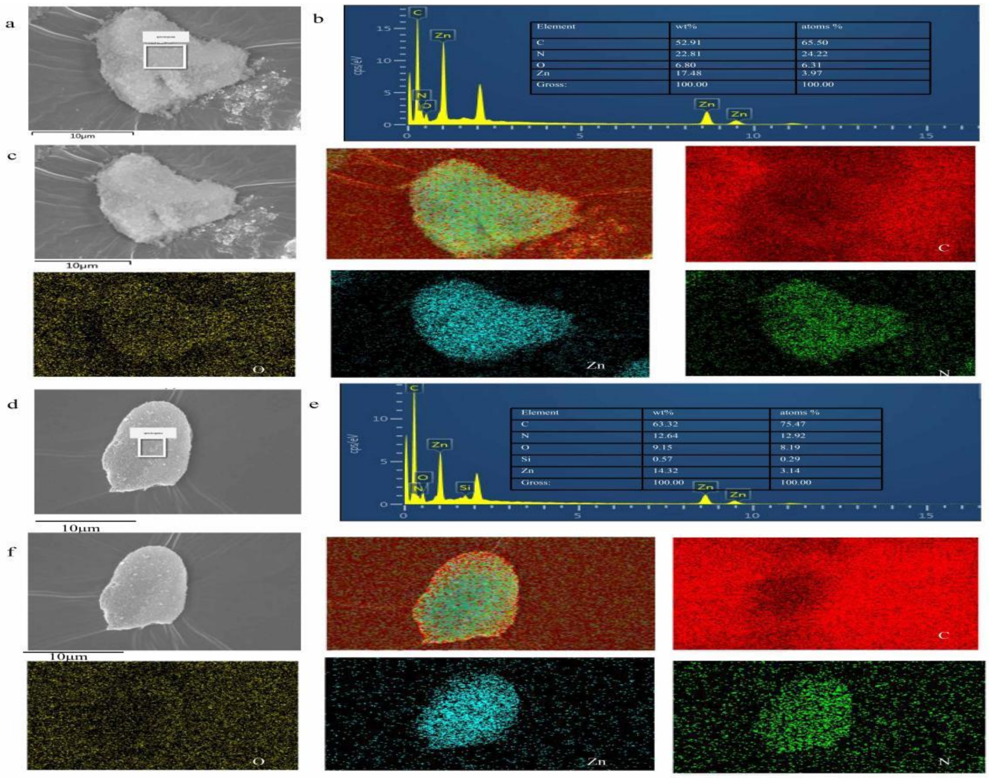

Figure S1. EDX analysis of ZIF-8/CEC@ZIF-8:(a) Electronic map of ZIF-8. (b) EXD images of ZIF-8. (c) EDX mapping of ZIF-8. Zn, C, O and N element.(d) Electronic map of CEC@ZIF-8. (e) EXD images of CEC@ZIF-8. (f) EDX mapping of CEC@ZIF-8. Zn, C, O and N element.

a

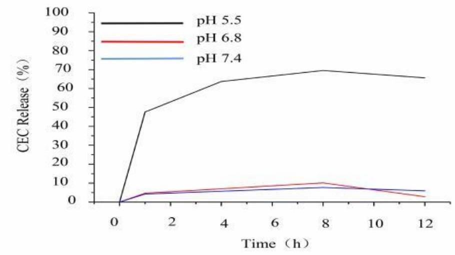

b

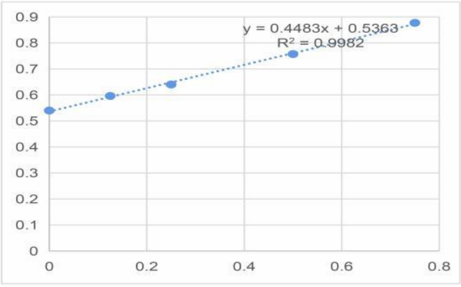

	CEC	CEC@ZIF-8
A(OD)	0.381	0.122
protein concentration (mg/ml）	1.5	0.121
DLC（wt%）		12.1
DLE（%）		44

Figure S2. Basic properties of CEC@ZIF-8. (a) CEC release profiles from CEC@ZIF-8 in PBS solution (pH = 5.5, 6.8 and 7.4) (b) Standard curve for the Bradford protein assay(left). DLC and DLE of CEC@ZIF-8 (right).

a

	PBS	5 μg/mL	10 μg/mL	50 μg/mL	100 μg/mL	150 μg/mL	200 μg/mL	1%Triton X-100	
CEC@ZIF-8 NPs	0.051	0.05	0.051	0.051	0.051	0.051	0.051	2.485	37℃, 30min
CEC@ZIF-8 NPs	0.05	0.049	0.05	0.05	0.05	0.05	0.05	2.095	37℃, 60min
CEC	0.05	0.05	0.05	0.051	0.051	0.051	0.051	2.614	37℃, 30min
CEC	0.05	0.051	0.051	0.05	0.05	0.051	0.051	2.488	37℃, 60min

b

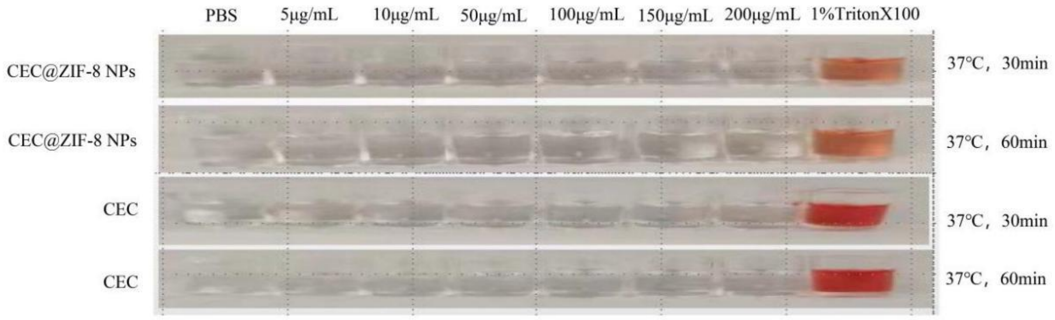

Figure. S3.Hemolytic assay for CEC@ZIF-8 NPs and free CEC samples at different concentrations in RBC. (a) OD value at 480nm and (b)Photographs of CEC@ZIF-8 NPs samples at different concentrations in RBC,with Triton (0.1%) as a positive control (100% hemolysis, the first from right) and PBS (pH 7.4) as a negative control (the first from left).

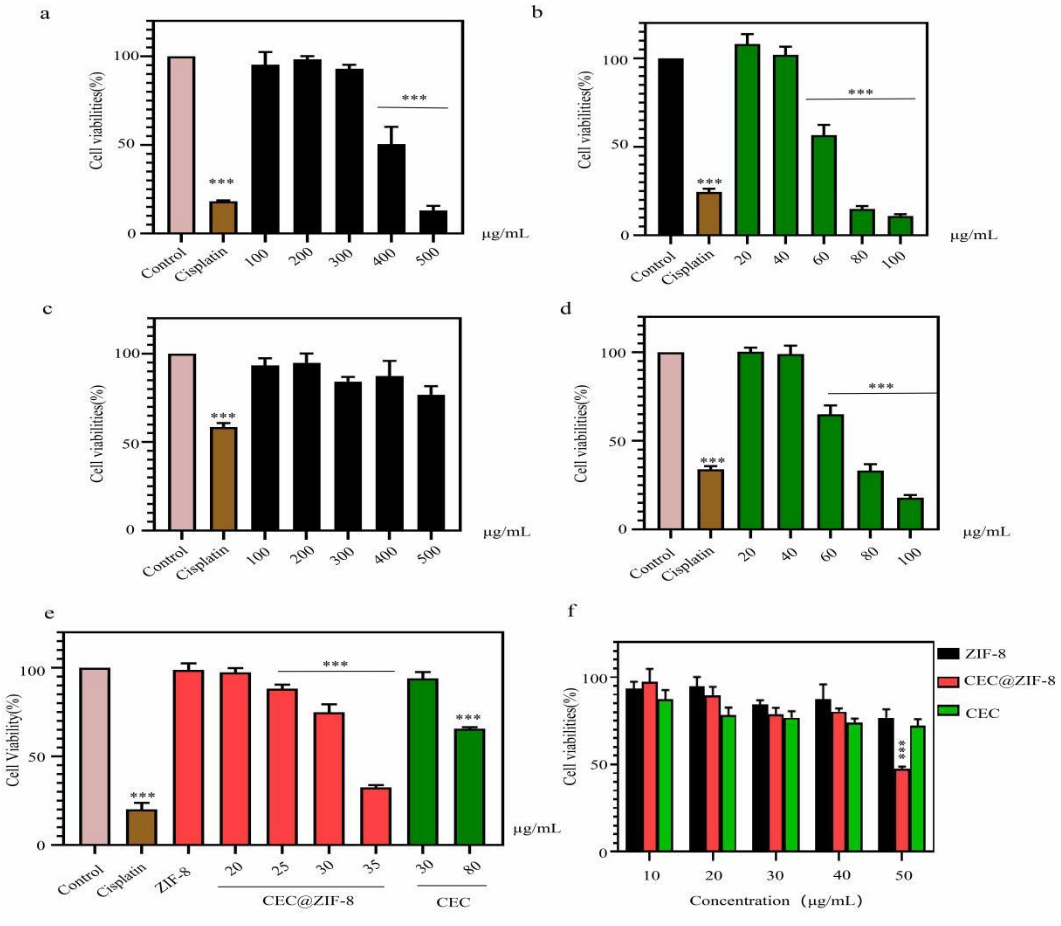

Figure. S4. In vitro cytotoxicity of ZIF-8 (a) and free CEC (b) against HeLa cells at different concentrations after incubation for 24 h.In vitro cytotoxicity of ZIF-8 (c) and free CEC (d) against SiHa cells at different concentrations after incubation for 24 h.(e) Cell viability of TC-1 cells after treatment with 1) free CEC, 2) CEC@ZIF-8 3) ZIF-8, for 24h.(f)Cell viability of SiHa cells after treatment with 1) free CEC, 2) CEC@ZIF-8 3) ZIF-8, for 24h.Compared to the Control group, ***< 0.001.

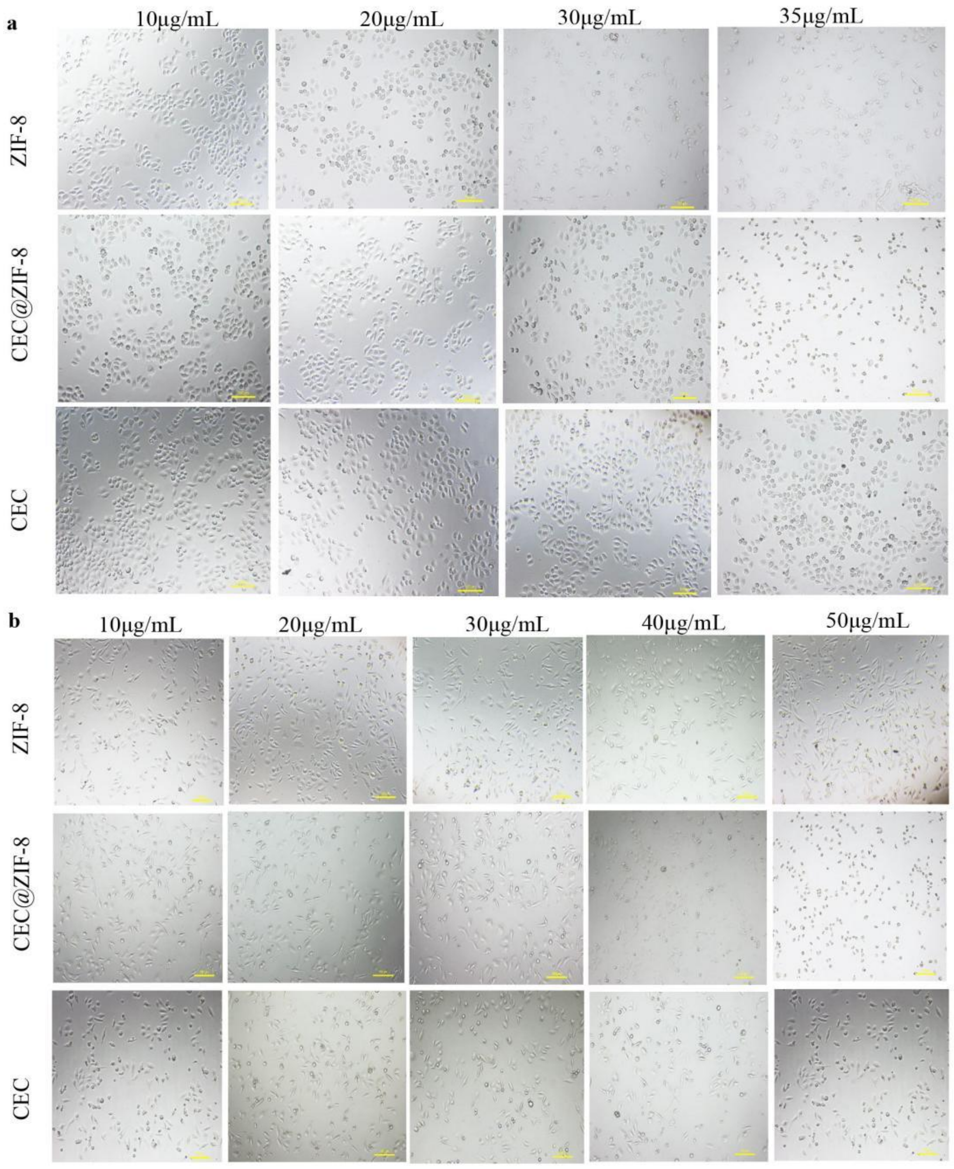

Figure S5. (a) Morphology of HeLa cells treated with different concentrations of ZIF-8, CEC@ZIF-8 and free CEC for 24h,(200×).(b) Morphology of SiHa cells treated with different concentrations of ZIF-8, CEC@ZIF-8 and free CEC for 24h,(200×).

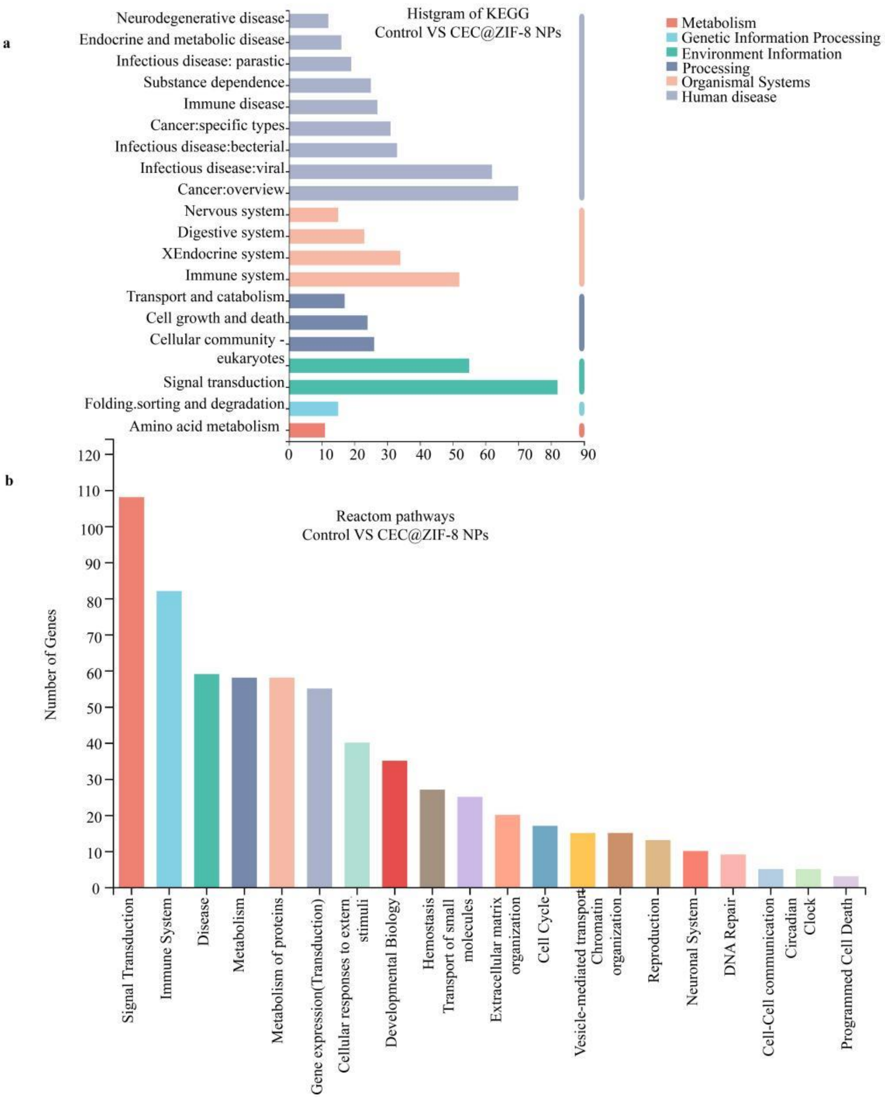

Figure S6. (a)The KEGG pathway of the difference gene is analyzed.(b)The Reactom pathway of the difference gene is analyzed.

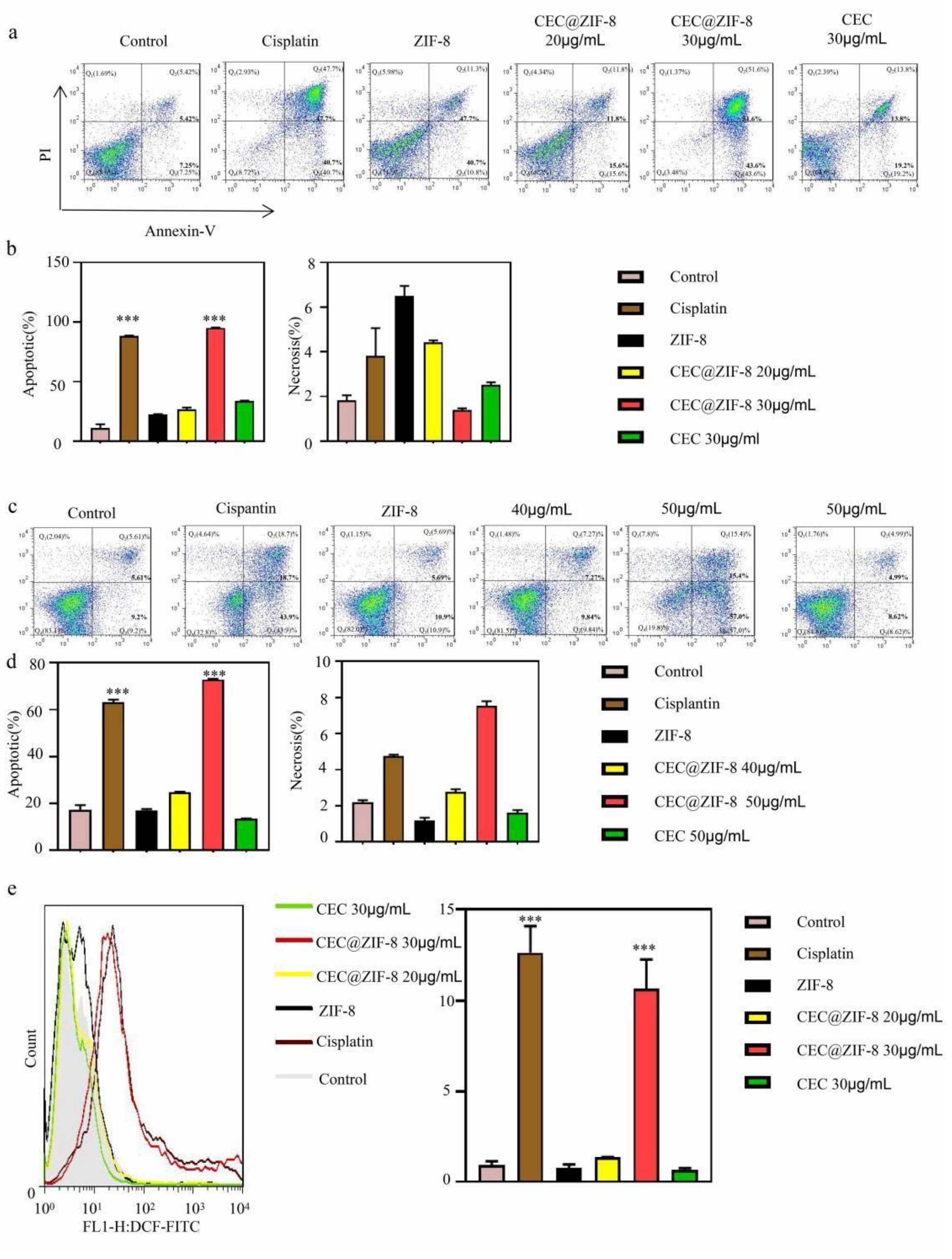

Figure S7.Anticancer action mechanism of CEC@ZIF-8 to cervical cancer cells.(a-b) TC-1 cells, (c-d) SiHa cells were treated with different concentrations of CEC@ZIF-8 NPs for 24h. The apoptosis and necrosis of cervical cancer cells were detected by flow cytometry.And statistical chart.(e) ROS production in TC-1 cells after CEC@ZIF-8 NPs treatment for 24 hours, the cells were stained with fluorescent probe DCFH-DA and analyzed by flow cytometry.And statistical chart.Compared to the Control group, *** $P < 0.001$.

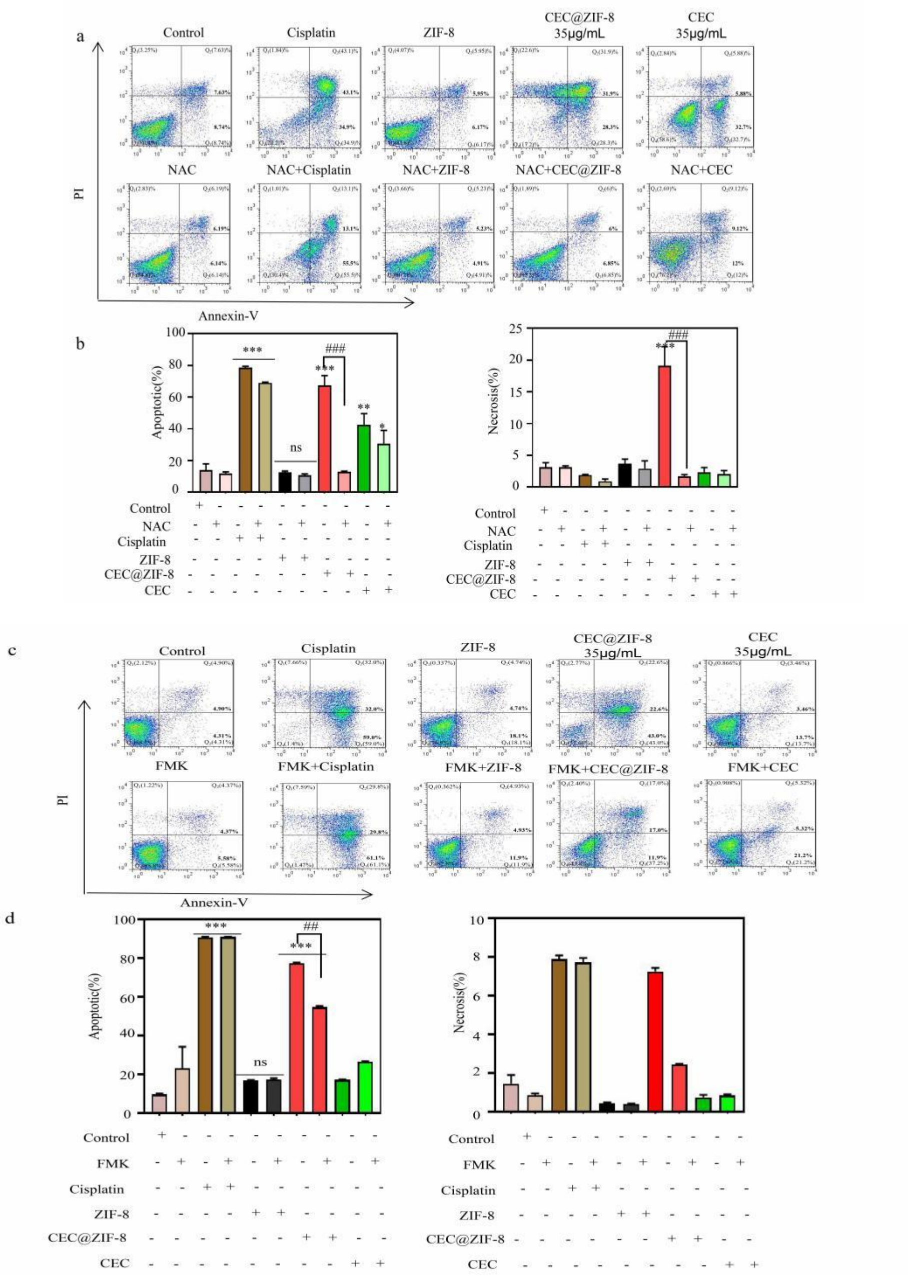

Figure S8.(a)After pretreatment of HeLa cells with 10 mM NAC for 1 h and treatment with CEC@ZIF-8 NPs and free CEC for 24 h for Annexin V/PI staining, the samples were analyzed by flow cytometry.(b) The statistical figure.(c) After pretreatment of HeLa cells with 10 mM FMK for 1 h and treatment with CEC@ZIF-8 NPs and free CEC for 24 h for Annexin V/PI staining, the samples were analyzed by flow cytometry.(d) The statistical figure.Compared to the Control group, *** P< 0.001.At the same concentration, the group treated with inhibitor was compared with the group without inhibitor treatment,##p<0.01,### p<0.001.

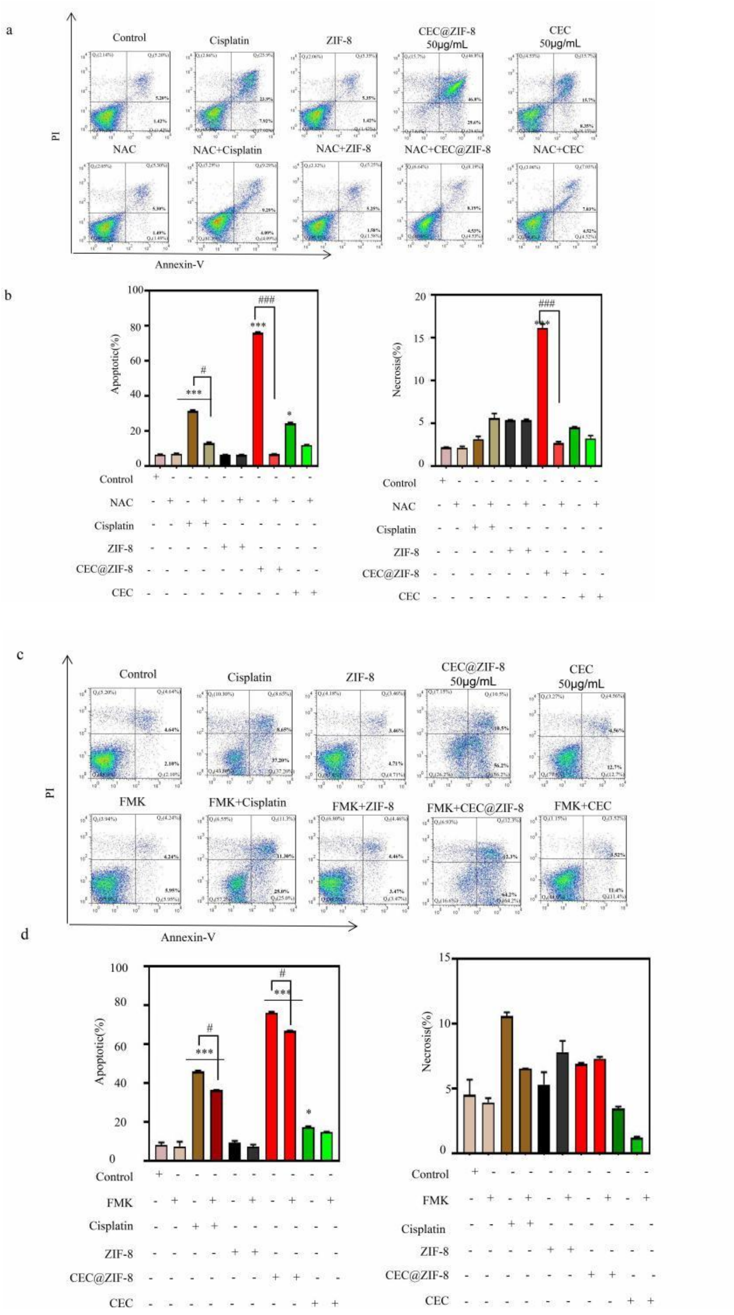

Figure S9(a)After pretreatment of SiHa cells with 10 mM NAC for 1 h and treatment with CEC@ZIF-8 NPs and free CEC for 24 h for Annexin V/PI staining, the samples were analyzed by flow cytometry.(b) The statistical figure.(c) After pretreatment of SiHa cells with 10 mM FMK for 1 h and treatment with CEC@ZIF-8 NPs and free CEC for 24 h for Annexin V/PI staining, the samples were analyzed by flow cytometry.(d) The statistical figure.Compared to the Control group, *** $P< 0.001$.At the same concentration, the group treated with inhibitor was compared with the group without inhibitor treatment,# $p<0.05$## $p<0.01$,### $p<0.001$.

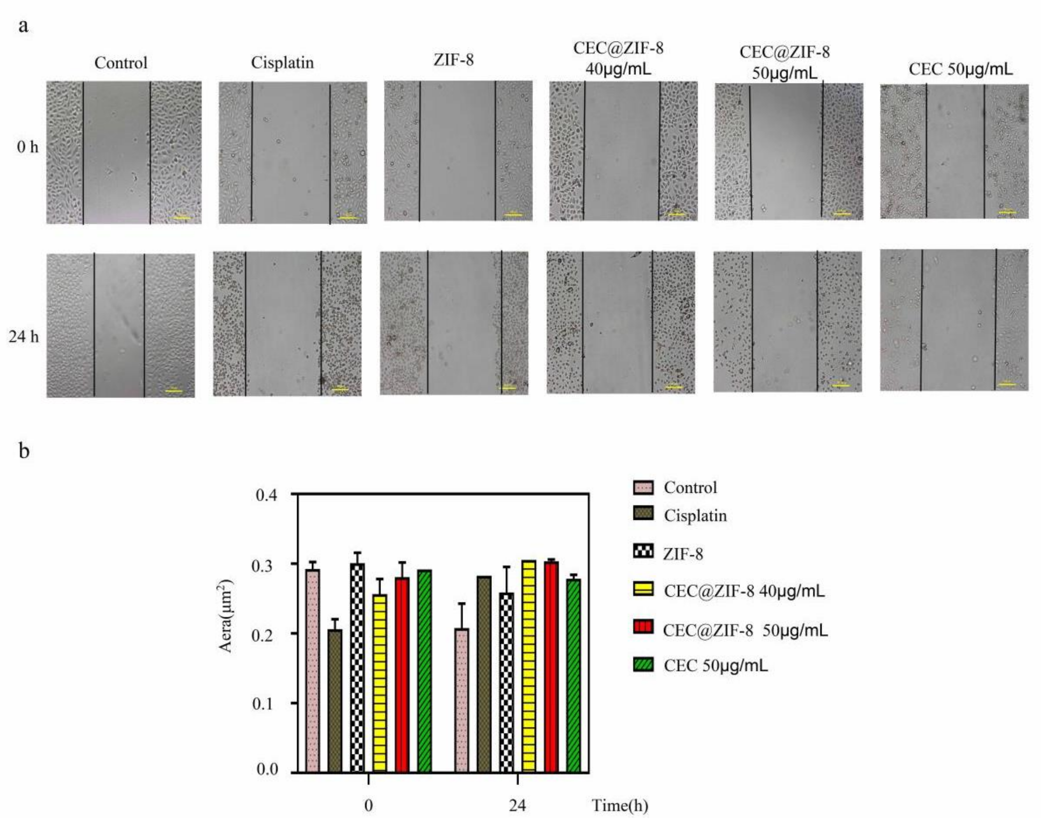

Figure S10.Effect of different concentrations of CEC@ZIF-8 nanoparticles on the migration of tumor cells.(a)SiHa cells were treated with different concentrations of CEC@ZIF-8 nanoparticles for 0h and 24h.(b) Statistical chart of migration ability of SiHa cells.

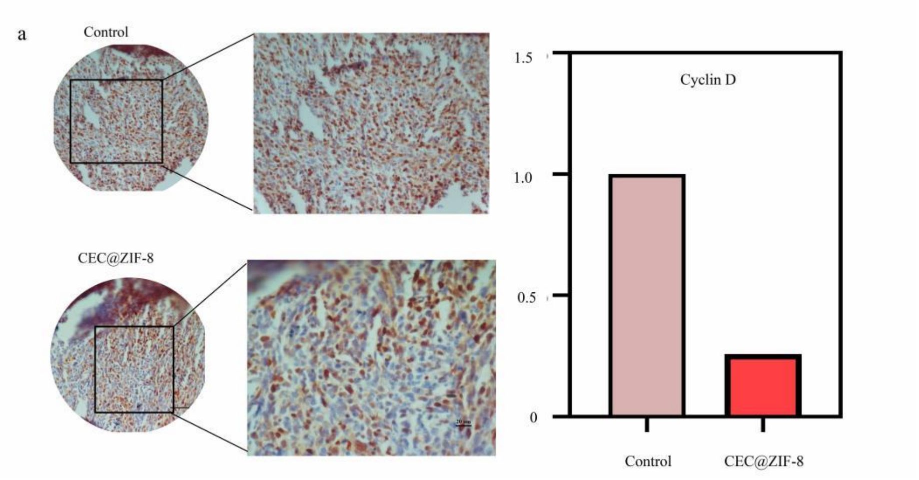

Figure S11. In vivo anticancer and Underlying mechanisms of CEC@ZIF-8.(a) Representative immunohistochemstry staining images (Cyclin D) of tumor slice after treatment. Scale bar refers to 20 μm.

a

	Control	Cisplatin	ZIF-8	CEC@ZIF-8	CEC
Spleen	12.29±4.31	3.95±1.06**	12.37±4.26	5.91±1.13	11.938±3.96
Thymus	3.31±0.62	2.43±1.38	2.56±0.44	2.58±0.43	2.15±1.66
Heart	5.56±0.60	7.89±1.28	6.13±0.46	6.01±0.12	6.10±1.06
Liver	65.72±4.44	61.58±3.20	61.61±3.93	69.58±4.46	70.71±4.37
Kidney	12.70±0.66	14.59±1.79	12.56±0.99	12.95±0.65	13.89±1.35
Lung	7.63±1.039	10.24±0.96	8.42±0.85	10.28±1.20	8.97±0.21

b

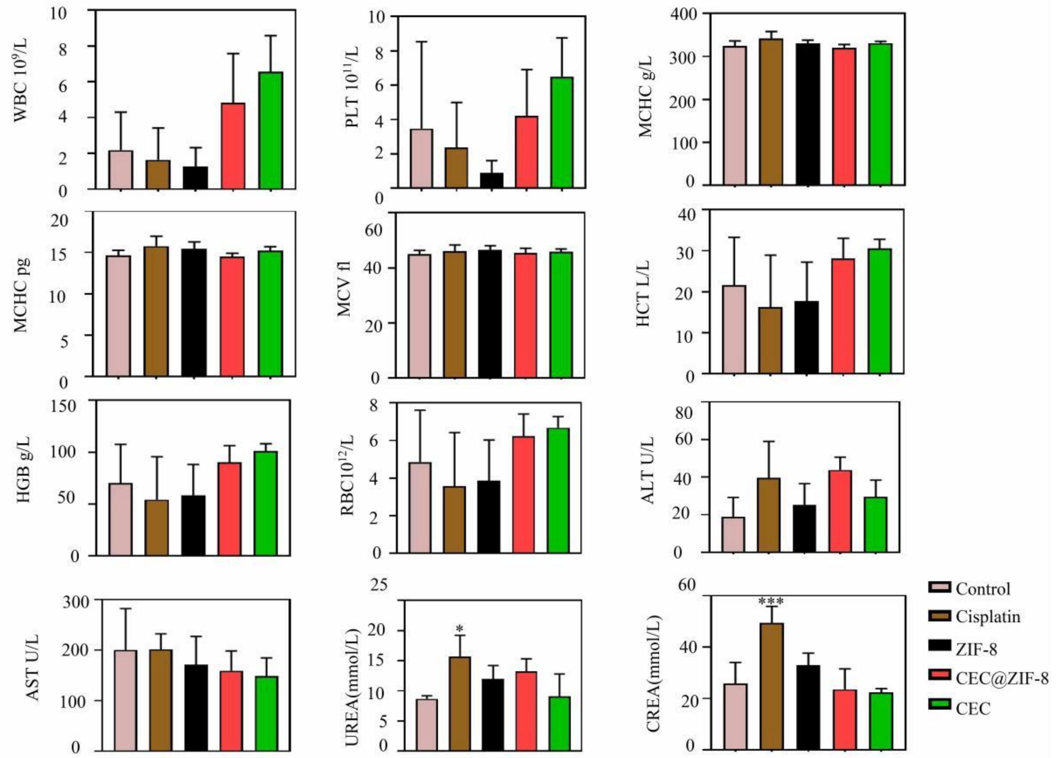

Figure S12. Safety evaluation of CEC@ZIF-8 in TC-1 tumor-bearing mice.(a) Effect of CEC@ZIF-8 on organ index of TC-1 tumor-bearing mice.(b) Blood biochemical indexes of TC-1 tumor-bearing mice after CEC@ZIF-8NPs treatment.Compared to the Control group, * $P<0.05$,** $P<0.01$,*** $P< 0.001$.
